# Unimolecular decomposition rates of a methyl-substituted Criegee intermediate *syn*-CH_3_CHOO†

**DOI:** 10.1039/d0ra01406k

**Published:** 2020-02-28

**Authors:** Yu-Lin Li, Mei-Tsan Kuo, Jim Jr-Min Lin

**Affiliations:** Institute of Atomic and Molecular Sciences, Academia Sinica Taipei 10617 Taiwan jimlin@gate.sinica.edu.tw; Department of Chemistry, National Taiwan University Taipei 10617 Taiwan

## Abstract

Criegee intermediates play important roles in atmospheric chemistry. Methyl Criegee intermediate, CH_3_CHOO, has two conformers, *syn*- and *anti*-conformers. *Syn*-CH_3_CHOO would undergo fast unimolecular decomposition to form OH radical *via* 1,4 H-atom transfer. In this work, unimolecular decomposition of *syn*-CH_3_CHOO was probed in real time with UV absorption spectroscopy at 278–318 K and 100–700 torr. We used water vapor as the scavenger of *anti*-CH_3_CHOO to distinguish the absorption signals of the two conformers. After removing the contributions from reactions with radical byproducts, reaction with water vapor and wall loss, we obtained the unimolecular reaction rate coefficient of *syn*-CH_3_CHOO (at 300 torr), which increases from (67 ± 15) s^−1^ at 278 K, (146 ± 31) s^−1^ at 298 K, to (288 ± 81) s^−1^ at 318 K with an Arrhenius activation energy of *ca.* 6.4 kcal mol^−1^ and a weak pressure dependence for 100–700 torr. Compared to previous studies, this work provides temperature dependent unimolecular rates of *syn*-CH_3_CHOO at higher pressures, which are more relevant to atmospheric conditions.

## Abstract 2

This work provides temperature dependent unimolecular rates of *syn*-CH_3_CHOO at higher pressures.

## Graphical abstract

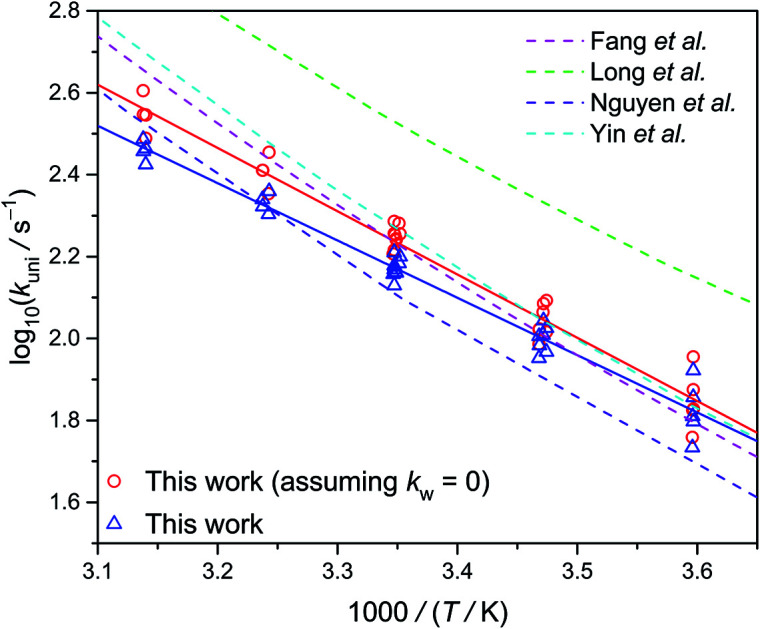

## Introduction

Criegee intermediates are very reactive carbonyl oxides formed in the reactions of ozone with alkenes (ozonolysis) and play important roles in atmospheric chemistry, including oxidation of water, SO_2_, NO_*x*_, *etc.* In particular, the decomposition of Criegee intermediates can be a significant source of OH radicals in the troposphere, especially during nighttime and winter.^1^

After the breakthrough of the efficient preparation of Criegee intermediates in 2012,^2^ the physical and chemical properties of simple Criegee intermediates have been widely investigated.^3,4^ Nowadays, it has been established that the reactivity of Criegee intermediates is highly structure-dependent.^4–6^ For example, CH_2_OO and *anti*-CH_3_CHOO react with water vapor very quickly but *syn*-CH_3_CHOO and (CH_3_)_2_COO react with water vapor much slower. On the other hand, *syn*-CH_3_CHOO and (CH_3_)_2_COO may undergo intramolecular H-atom transfer from the methyl group to the terminal oxygen atom, resulting in OH production.^7,8^

However, there have been large discrepancies in the unimolecular (also bimolecular and termolecular) reaction rates of Criegee intermediates. If we focus on the unimolecular rate coefficients of *syn*-CH_3_CHOO reported after 2012, the reported experimental value^9–12^ at 298 K ranges from 3 to 300 s^−1^ whilst the reported theoretical value^13–15^ is from 24 to 330 s^−1^.^1^ Unimolecular rates may control the steady-state concentrations and thus the impact of such a Criegee intermediate in the atmosphere. It is important to have reliable experimental data at atmospherically relevant pressure and temperature.

Lester and coworkers excited *syn*-CH_3_CHOO, *syn*-C_2_H_5_CHOO and (CH_3_)_2_COO to a high vibrational energy *via* infrared laser and monitored the OH production by laser-induced fluorescence. The OH formation rates have been measured at specific vibrational energies. Master equation modeling based on these microcanonical rates and high-level quantum chemistry calculations has been used to estimate the thermal decay rate coefficients at 298 K to be 166/122 s^−1^ for *syn*-CH_3_CHOO,^14,16^ 279, and 276 s^−1^ for *syn*-C_2_H_5_CHOO^17^ and (CH_3_)_2_COO,^14^ respectively.^18^ The fast rates indicate thermal decomposition is the main sink for these Criegee intermediates in the atmosphere.

Zhou *et al.* have monitored the OH products of *syn*-CH_3_CHOO in a flow cell. The thermal decomposition rate at 298 K (25 to 100 torr) is reported to be 182 ± 66 s^−1^.^19^ Their results also support that the thermal decomposition is the main atmospheric process for *syn*-CH_3_CHOO.

In this work, we used water vapor to scavenge *anti*-CH_3_CHOO^20^ and directly monitored the kinetics of *syn*-CH_3_CHOO *via* its strong UV absorption in real time.^21^ The unimolecular rates were determined at atmospherically relevant temperatures and pressures. The results are compared with previous experimental and theoretical works.

## Experimental method

Following previous studies, we used CH_3_CHI_2_ (Aldrich, ≥98%) as the precursor and generated CH_3_CHI radicals by applying photolysis laser (Excimer laser, KrF 248 nm, Coherent COMPexPro 205F). Under high [O_2_] (10 torr), the iodo-radicals would react with O_2_ rapidly to form CH_3_CHOO.^12^ The photolysis laser beam was coupled into the reactor by a long-pass filter (275 nm, Eksma Optics, customized). After passing through the reactor, the photolysis beam was reflected out by another long-pass filter, and the laser pulse energy was monitored with an energy meter (Gentec QE25SP-H-MB-D0). Continuous UV probe beam originated from a broadband Xe lamp (Energetiq, EQ-99) went through the reactor six times. The probe beam overlapped collinearly with the photolysis beam, resulting in an effective absorption path length of *ca.* 426 cm.

The probe wavelength was selected by a band-pass filter (340 nm, 10 nm bandwidth, OD4, Edmund #65129). The time-dependent absorption change was detected with a balanced photodiode detector (Thorlab PDB450A) and recorded in real time with a high-definition oscilloscope (LeCroy HDO4034, 4096 vertical resolution). All time traces were averaged for *ca.* 120 laser shots to increase S/N ratio. We found a small absorption change even without adding the precursor, which was caused by the photolysis laser and the long-pass filters. This background could be subtracted by recording background traces before and after each experimental set. All reported time traces have undergone background subtraction.

CH_3_CHI_2_ vapor was carried by a stream of N_2_. All the gas flows were controlled by mass-flow controllers (Brooks, 5850E or 5800E). A small portion of CH_3_CHI_2_ vapor was introduced into a residual gas analyzer (SRS RGA200) to monitor the impurity. Most of CH_3_CHI_2_ vapor would mix with O_2_ and then flowed into an absorption cell (90 cm in length) to measure the UV absorption spectrum with a deuterium lamp (Hamamatsu L10904) and a mini spectrometer (Ocean Optics USB-2000). The water vapor concentration was controlled by varying the ratio of dry and moisturized N_2_ gases and monitored with a humidity sensor (Rotronic HC2-S, 0.8% RH accuracy at room temperature). The water vapor was pre-cooled or pre-heated by flowing through a copper tube (1/4′′ OD, *ca.* 75 cm long) which was immersed in a circulating water bath. Two streams of the gas mixtures (water vapor/N_2_ and precursor/O_2_) were mixed together right before entering the reactor. The laser repetition rate was set to *ca.* 1 Hz to fully refresh the gas between consecutive laser shots.

The temperature of the water-jacketed reactor was controlled (±0.5 K) by using a circulating water bath (Yih Der BL-730), and detected by three resistance temperature detectors (RTD, Newport Omega F2020-1000-A). The pressures at the water reservoir, absorption cell and reactor were measured by diaphragm gauges (1000 torr, INFICON, ±0.2% of reading).

## Results

We synthesized CH_3_CHOO (a mixture of *anti* and *syn* conformers) *via* a well-known method.^7,12^

R1 CH_3_CHI_2_ + *hν* → CH_3_CHI + I

R2 CH_3_CHI + O_2_ → CH_3_CHOO + I

R3 CH_3_CHI + O_2_ + M → CH_3_CHIOO (adduct) + M

Similar to the case of preparing CH_2_OO, adduct formation (R3) is also expected and would reduce the yield of CH_3_CHOO at high pressures.^22,23^

### Removing the contribution of *anti*-CH_3_CHOO

**Fig. 1:**
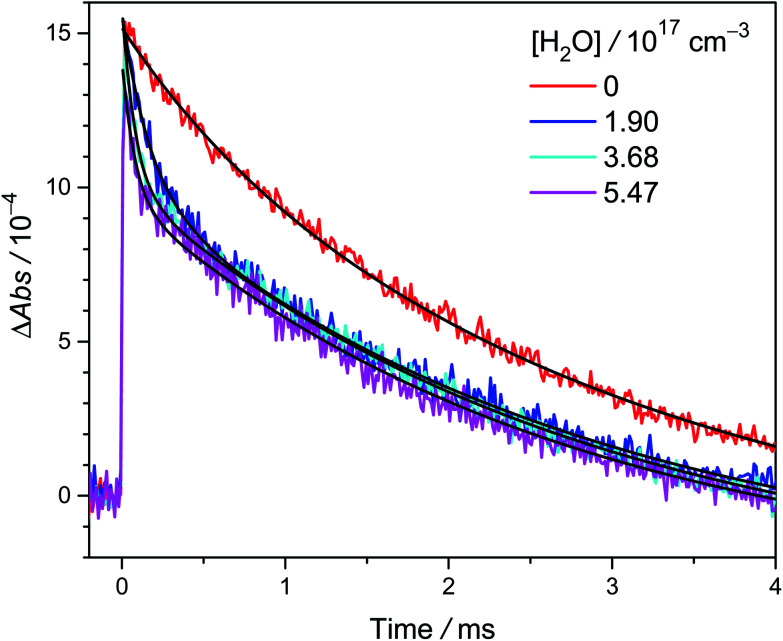
Typical time traces of CH_3_CHOO absorption probed at 340 ± 5 nm under various [H_2_O] (308 K and 305 torr, Exp. W2-3: [CH_3_CHI_2_] = 1.4 × 10^14^ cm^−3^, laser fluence = 1.7 mJ cm^−2^, see Table S1† for details). The photolysis laser pulse sets the time zero. The black lines are two-exponential fit to the data (see text). The fast decay signal is assigned to *anti*-CH_3_CHOO, the slow one to *syn*-CH_3_CHOO.

Fig. 1 shows typical time traces of CH_3_CHOO absorption signals recorded at 340 nm under various [H_2_O]. We can see that the fast component decays faster at higher [H_2_O], while the effect of [H_2_O] on the slow component is rather weak. Because *anti*-CH_3_CHOO reacts with water vapor much faster than *syn*-CH_3_CHOO does,^20^ we may separate their signals by fitting the time traces with two exponential functions (eqn (1)).1
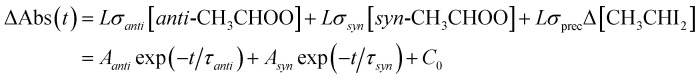
where *L* is the absorption path length; *σ*_*anti*_, *σ*_*syn*_ and *σ*_prec_ are the absorption cross sections of *anti*-CH_3_CHOO, *syn*-CH_3_CHOO, and the CH_3_CHI_2_ precursor, respectively; *A*_*anti*_ and *A*_*syn*_ are the signal amplitudes for *anti*-CH_3_CHOO and *syn*-CH_3_CHOO, respectively; *τ*_*anti*_ and *τ*_*syn*_ are the corresponding lifetimes; *C*_0_ accounts for the depletion of the precursor which has a weak absorption at 340 nm (1.07 × 10^−18^ cm^2^).^24^ In further analysis, we have *k*_obs_ = *τ*_*syn*_^−1^ and [*syn*-CH_3_CHOO]_0_ = *A*_*syn*_/(*Lσ*_*syn*_).

The fast kinetics of *anti*-CH_3_CHOO reaction with water vapor has been investigated in depth by a few groups.^12,20,25^ Therefore we do not repeat it here. Water vapor efficiently scavenges *anti*-CH_3_CHOO; Fig. 1 shows that at [H_2_O] > 1.9 × 10^17^ cm^−3^, only *syn*-CH_3_CHOO signal remains for *t* > 1 ms. The reaction of *syn*-CH_3_CHOO with H_2_O has been reported to be much slower than that of *anti*-CH_3_CHOO.^12,13,20,25^ More kinetic investigation on the reaction of *syn*-CH_3_CHOO with H_2_O will be discussed later in this paper.

### Effect of bimolecular reactions of *syn*-CH_3_CHOO with radical byproducts

**Fig. 2:**
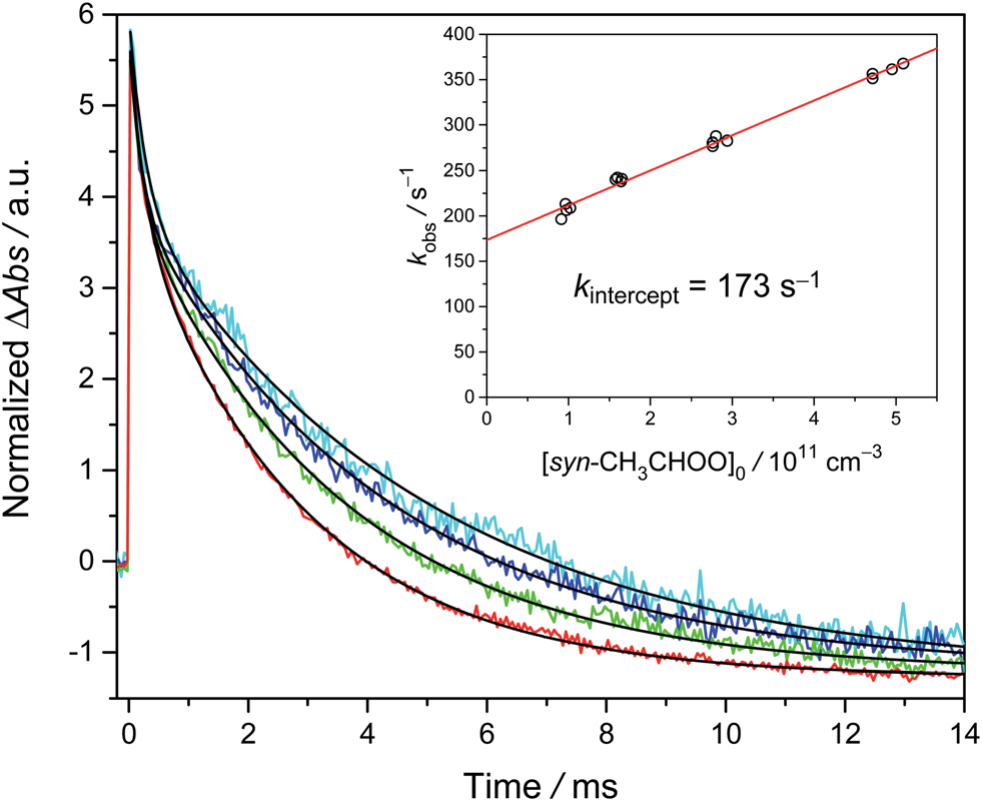
Typical time traces of CH_3_CHOO probed at 340 ± 5 nm at [H_2_O] = 1.9 × 10^17^ cm^−3^; different colors denote various precursor concentrations [CH_3_CHI_2_]_0_ (4.8 × 10^13^ cm^−3^ (cyan), 7.7 × 10^13^ cm^−3^ (blue), 1.3 × 10^14^ cm^−3^ (green), and 2.5 × 10^14^ cm^−3^ (red)). These traces were acquired at 299 K and 301 torr with a laser fluence of 1.7 mJ cm^−2^ (Exp. 2-1, see Table S2†). For each trace, the black line is the two-exponential fit (eqn (1)) to the signal of CH_3_CHOO. The fast decay signal is assigned to *anti*-CH_3_CHOO; the slow one is assigned to *syn*-CH_3_CHOO. The slightly negative baseline is due to the depletion of the precursor CH_3_CHI_2_, which is a constant in the detection time window and does not affect the kinetics. We scaled the peak absorbance of *syn*-CH_3_CHOO to the same value for easier visualization. Inset: the plot of *k*_obs_*versus* [*syn*-CH_3_CHOO]_0_. The red line is the linear fit.

Fig. 2 shows the time traces of CH_3_CHOO absorption signals recorded under various precursor concentrations [CH_3_CHI_2_]_0_ and a fixed photolysis laser fluence. In this experiment, we added water ([H_2_O] = 1.9 × 10^17^ cm^−3^) to scavenge *anti*-CH_3_CHOO and the absorption signal of the remaining *syn*-CH_3_CHOO predominates in the time traces for *t* > 1 ms. We can see that the lifetime of *syn*-CH_3_CHOO becomes shorter at higher [CH_3_CHI_2_]_0_. This is mainly due to the reactions of *syn*-CH_3_CHOO with radical byproducts like iodine atoms, similar to previous results for CH_2_OO,^23^*syn*-CH_3_CHOO^19,26^ and (CH_3_)_2_COO.^27^ The inset of Fig. 2 shows the observed decay rate coefficient *k*_obs_ of *syn*-CH_3_CHOO plotted against its fitted peak height [*syn*-CH_3_CHOO]_0_, which was estimated by using the Beer–Lambert law with a reported cross section *σ*_*syn*_ = 1.19 × 10^−17^ cm^2^ at 340 nm (ref. 21) (which is consistent with the results by Sheps *et al.*^25^) and effective length *L* = 426 cm.

Under our experimental conditions, the concentrations of the radical byproducts (I, CH_3_CHOO, CH_3_CHIOO, *etc.*) are proportional to [*syn*-CH_3_CHOO]_0_. The related chemical reactions include (R1)–(3). Thus, we can use [X], [X] ∝ [*syn*-CH_3_CHOO]_0_, to represent the effective concentration of the total radical species. We used the following kinetic model to analyze the experimental data.

2 *k*_obs_ = *k*_uni_ + *k*_X_[X] + *k*_w_[H_2_O] + *k*_wall_

where *k*_uni_ is the unimolecular decomposition rate coefficient; *k*_X_ is the effective rate coefficient for the reaction of *syn*-CH_3_CHOO with total radicals X; *k*_w_ and *k*_wall_ represent the rate coefficients for the reaction of *syn*-CH_3_CHOO with H_2_O and the wall loss, respectively. The linear behavior of *k*_obs_ against [*syn*-CH_3_CHOO]_0_ is consistent with the above model.

**Fig. 3:**
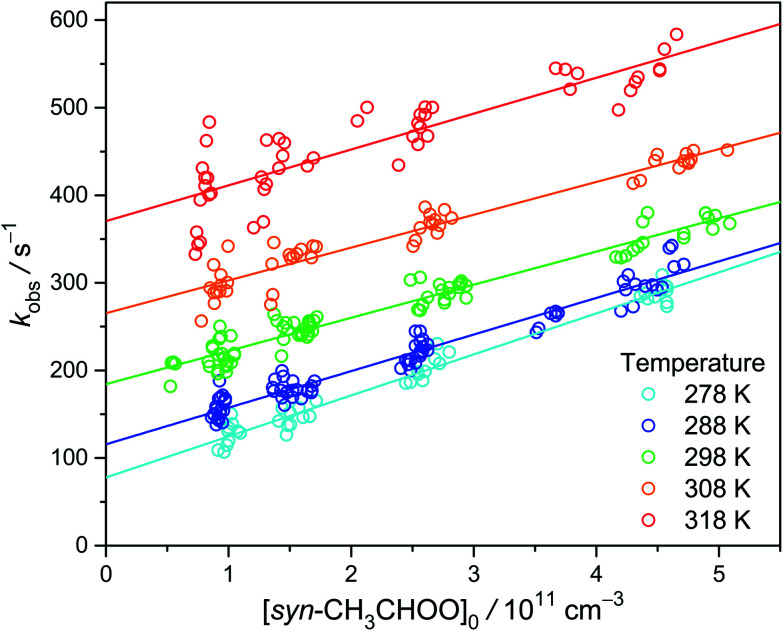
Plot of *k*_obs_ as a function of [*syn*-CH_3_CHOO]_0_ at different temperatures. The experiments were conducted at 300 torr and detailed experimental conditions are shown in Table S2.†

Similar experiments have also been performed at temperatures from 278 K to 318 K. The results are shown in Fig. 3. Again, the positive slope indicates the observed decay rate coefficient becomes larger at higher radical concentrations. Remarkably, the intercept rate is substantially larger at higher temperature, suggesting that the unimolecular rate is faster at higher temperature.

### Effect of water vapor

**Fig. 4:**
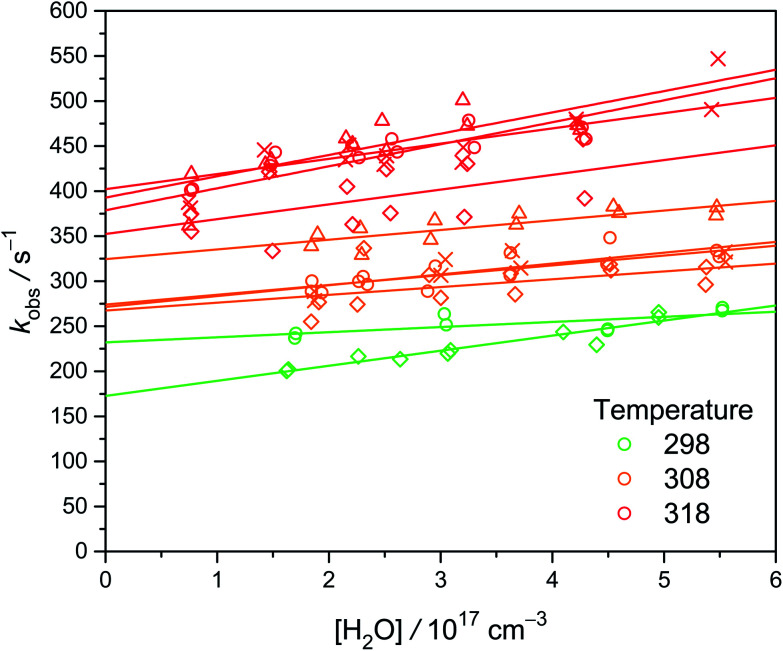
Plot of *k*_obs_ as a function of [H_2_O] at three temperatures (color-coded) under 300 torr. Different symbols represent different sets of experiments at various [CH_3_CHI_2_]_0_, while each set of experiment was conducted at a constant laser power (see Table S1†). The lines are linear fit to the data. Larger values of the intercept rates are due to higher temperature or higher [CH_3_CHI_2_]_0_.

We observed that the decay rate of *syn*-CH_3_CHOO becomes slightly faster at higher [H_2_O], especially at higher temperatures (see Fig. 4). However, the slopes are not very significant, especially considering the large scatters of the data. The obtained slopes (*k*_w_) at 298 K and 300 torr are on the order of 1 × 10^−16^ cm^3^ s^−1^ (Exp. W3-1 and W4-1, see Table S1†), much larger than the theoretical results ranging from 1.9 × 10^−19^ to 2.4 × 10^−18^ cm^3^ s^−1^.^13,28^ The source of this discrepancy is not fully clear. It is likely that minor impurity in the water vapor had some effect (different sets of gas tubing were used in Experiments W3 and W4†). It is difficult to determine the small rate coefficient of *syn*-CH_3_CHOO reaction with water; as evidenced by previous investigations by Sheps *et al.*^25^ and Taatjes *et al.*^12^ which only reported upper limits of 2 × 10^−16^ and 4 × 10^−15^ cm^3^ s^−1^, respectively, for the rate coefficient. A similar difficulty has also been reported for the case of (CH_3_)_2_COO reaction with water (*k*_w_ < 1.5 × 10^−16^ cm^3^ s^−1^).^29^ Fig. S3† shows the Arrhenius plot of *k*_w_. Because we cannot measure *k*_w_ at temperatures lower than 298 K, the values of *k*_w_ from the Arrhenius fit are used. Considering the difficulties in the experiments, here we report a conservative upper limit of 1 × 10^−16^ cm^3^ s^−1^ for *k*_w_ at 298 K.

### Effect of wall loss

**Fig. 5:**
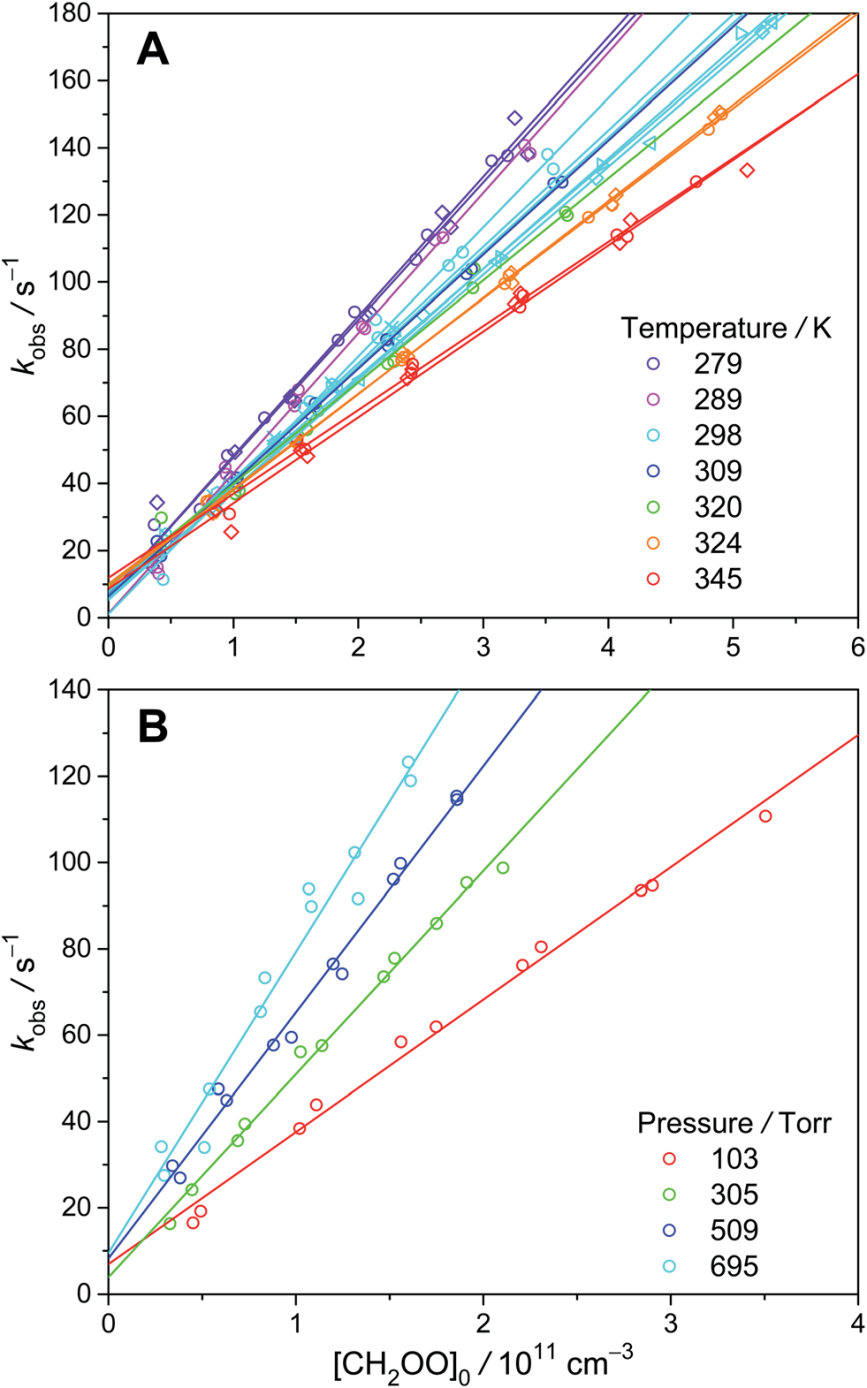
*k*_obs_ as a function of [CH_2_OO]_0_ at various temperatures (A) and pressures (B) (color-coded). Different symbols represent different experimental sets.

To estimate the wall loss in our system, we measured the decay rates of CH_2_OO under similar experimental conditions. The thermal decomposition of CH_2_OO has been calculated to be quite slow.^6^ In addition, Berndt *et al.* have performed time-resolved experiments of C_2_H_4_ ozonolysis, which yielded a rate coefficient of (0.19 ± 0.07) s^−1^ for the unimolecular reaction of CH_2_OO at 298 K and 1 bar.^30^ Furthermore, Stone *et al.* directly measured the unimolecular reaction of CH_2_OO at higher temperatures (450–650 K) and obtained a rate coefficient of (1.1^+1.5^_−1.1_ × 10^−3^) s^−1^ at 298 K and 760 torr using a master-equation method.^31^ Thus, the observed CH_2_OO decay (*k*_obs_, see Fig. 5) in our system should mainly come from the wall loss (intercept) and bimolecular reactions (slope). As shown in Fig. S6,† the results at different temperatures and pressures are quite similar, with an average value of about 9 s^−1^ for *k*_wall_. At our pressure range, diffusion is too slow to explain the observed wall loss rate; gas turbulence, which is expected to be more severe at higher pressures, should be the main cause of the observed wall loss (see ESI† for details). As gas turbulence would not be affected by the highly diluted precursor molecules and Criegee intermediates, we assume the wall loss rate of *syn*-CH_3_CHOO is the same as that of CH_2_OO.

### *k*_uni_ and its temperature dependence

Here we give one example of determining the unimolecular rate at 298 K.

2′ *k*_uni_ = *k*_obs_ − *k*_X_[X] − *k*_w_[H_2_O] − *k*_wall_ = *k*_intercept_ − *k*_w_[H_2_O] − *k*_wall_

In Fig. 2 inset, we can see that the *k*_X_[X] term has a significant contribution to *k*_obs_. This contribution can be removed by extrapolating *k*_obs_ to zero radical concentration (*i.e.*, using *k*_intercept_). For Exp. 2-1 (299 K, 301 torr, see Table S2†), *k*_intercept_ = 173 s^−1^, *k*_w_[H_2_O] = 17 s^−1^, *k*_wall_ = 9 s^−1^ and we have *k*_uni_ = 173 − 17 − 9 = 147 s^−1^. See ESI† for details.

**Fig. 6:**
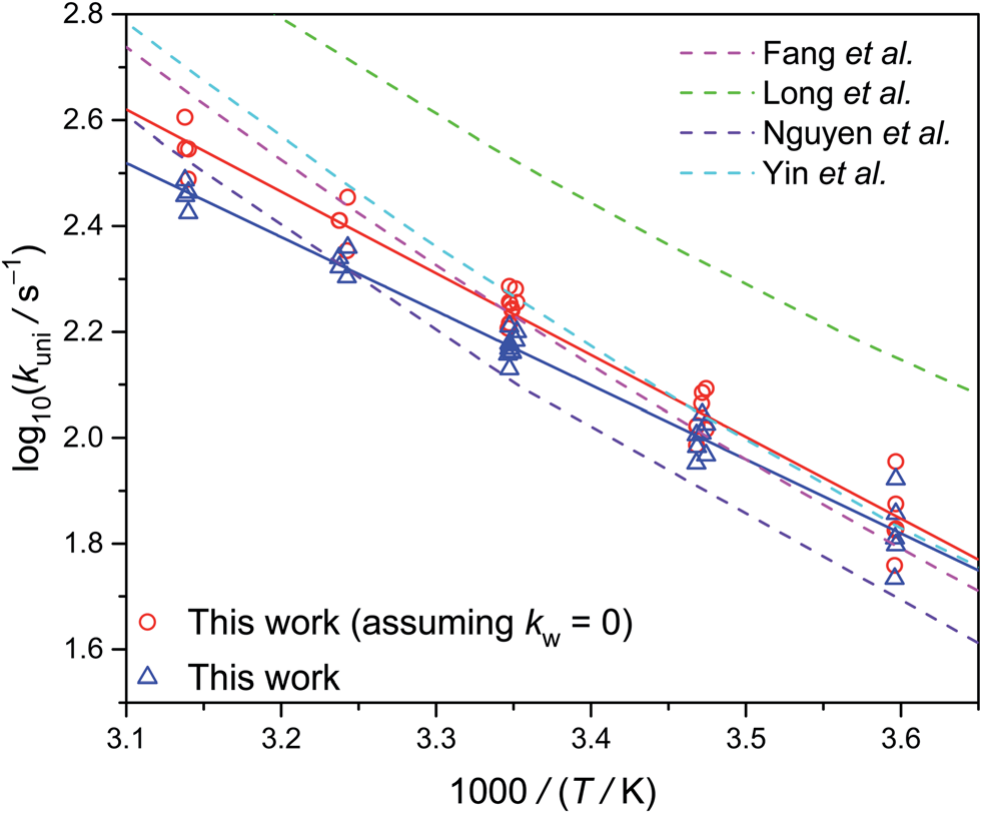
Arrhenius plot of the thermal decomposition rate coefficient of *syn*-CH_3_CHOO at 300 torr (Table S2†). The dashed lines are theoretical data reported by various groups at the high pressure limit.^13–15,32^ The experimental data shown as triangles and circles represent the data with and without subtracting the water contribution, respectively.

Fig. 6 shows the Arrhenius plot of *k*_uni_ measured at 300 torr, as well as a few theoretical results. Our data are consistent with the theoretical results, especially those by Fang *et al.*^14^ and Yin *et al.*^15^ However, the theoretical slopes (activation energy) are all a bit higher than the experimental value. The reason for this difference is unclear. As mentioned above, the water reaction rates *k*_w_[H_2_O] are difficult to quantify. If we assume *k*_w_ = 0, the resulted activation energy would be a bit closer to the theoretical values (see Table 1).

**Table 1:** Thermal decomposition rate coefficient and Arrhenius activation energy of *syn*-CH_3_CHOO

	*k*_uni_/s^−1^	*E*_a_/kcal mol^−1^
This work	146 ± 31^a^	6.4 ± 0.2^b^
This work (assuming *k*_w_ = 0)	173 ± 31^a^	7.1 ± 0.3^b^
Fang *et al.*^14^	166^c^	8.5 ± 0.1^b^
Long *et al.*^13^	328^c^	7.7 ± 0.2^b^
Nguyen *et al.*^32^	124^c^	8.2 ± 0.4^b^
Yin *et al.*^15^	182^c^	8.6 ± 0.2^b^

a At 298 K and 300 torr. The error bar is one standard deviation (see ESI).

b The activation energy (including theoretical ones) is obtained from the slope of the linear fitting of log(*k*) against 1/*T* within 278–318 K; the error bar is one standard deviation of the fitting.

c Theoretical result at 298 K at the high pressure limit.

### Pressure dependence of *k*_uni_

**Fig. 7:**
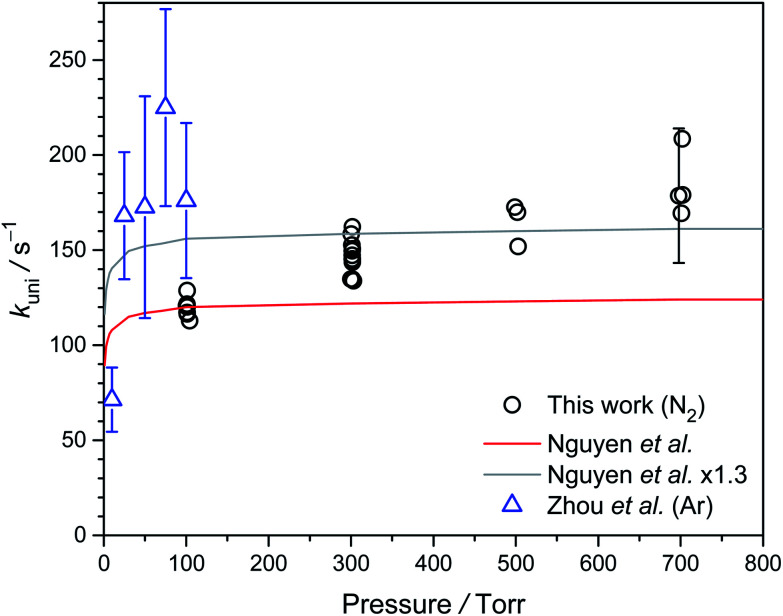
Plot of *k*_uni_ of *syn*-CH_3_CHOO against pressure from 100 to 700 torr at 298 K (Table S3†). The experimental data reported by Zhou *et al.* are shown as blue triangles,^19^ and the theoretical data reported by Nguyen *et al.* is shown as a red line.^32^ In order to compare with the experimental data, we also scaled the data of Nguyen *et al.* by a factor of 1.3 (gray line).

Fig. 7 shows the pressure dependence of *k*_uni_ at 298 K. Because we used water to scavenge *anti*-CH_3_CHOO, we cannot measure *k*_uni_ at pressure lower than 100 torr. Our data from 100 to 700 torr suggest slightly higher unimolecular rate at higher pressure, albeit some scattering of the data points. The low-pressure results of Zhou *et al.* are also plotted in Fig. 7. As will be discussed below, Zhou *et al.*^19^ may have underestimated their diffusion loss and thus overestimated their *k*_uni_ values, especially for data at lower pressures. Overall, the observed pressure dependences are roughly consistent with the theoretical trend.

## Discussion

### Effect of second-order reactions

In previous works on the thermal decomposition reactions of (CH_3_)_2_COO by Smith *et al.*^27^ and of *syn*-CH_3_CHOO by Zhou *et al.*,^19^ the authors have considered the contributions of second-order reactions (including the reaction with iodine atoms and self-reaction of Criegee intermediates^26^). Following the notation of Smith *et al.*, the corresponding kinetic equation can be expressed as below.^27^3
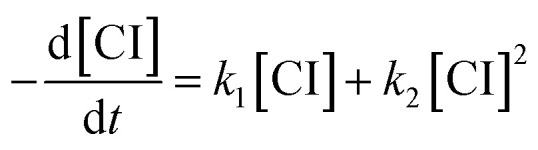
where [CI] stands for the concentration of the Criegee intermediate of interest. However, Smith *et al.* have also demonstrated that after extrapolation to zero concentration, a simplified model of pseudo-first-order reaction would give essentially the same results for the intercept rates.^27^ That is, we may use eqn (4), if [CI]_0_ is low.4
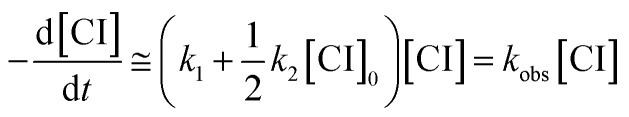


As mentioned by Smith *et al.*, the difference between the two models only shows up at high concentration data.^27^ As shown in Fig. S5,† the simulated time traces of the solutions of eqn (3) and (4) are quite similar for [CI]_0_ = 3 × 10^11^ cm^−3^, *k*_1_ = 150 s^−1^, *k*_2_ = 1.6 × 10^−10^ cm^3^ s^−1^. Therefore, we chose to use eqn (4) for more efficient data analysis.

### Tunneling and potential energy surface

Lester, Klippenstein and coworkers have shown that H-atom tunneling is the main mechanism of the unimolecular decay of *syn*-CH_3_CHOO.^14,16,33^ Green *et al.* of the same group further investigated the tunneling dynamics by using *syn*-CD_3_CHOO sample.^34^ Their theoretical calculation describes the energy-specific unimolecular rates quite well. Based on these successes, they can further estimate the unimolecular rates under thermalized conditions. The result is plotted in Fig. 6 (Fang *et al.*), which agrees with our experimental data. The subtle difference in the slopes of the Arrhenius plots may be due to minor differences in (i) potential energy surface, (ii) tunneling model, and (iii) experimental uncertainties.

### Compare to the recent results of Zhou *et al.*

Recently Zhou *et al.* measured the unimolecular rates of *syn*-CH_3_CHOO by probing the OH products with laser-induced fluorescence.^19^ Because the barrier of OH production of *anti*-CH_3_CHOO is much higher than that for *syn*-CH_3_CHOO,^13,15^ Zhou *et al.* do not need to scavenge *anti*-CH_3_CHOO.

Their measurements were under 10 to 100 torr,^19^ limited by the lower sensitivity of the OH laser-induced fluorescence at higher pressure. Our work provides data at higher pressures up to 700 torr, much more relevant to the atmospheric conditions. In addition, perhaps due to sensitivity issue, Zhou *et al.* used higher concentrations of the Criegee intermediate in their experiments (judged by the faster observed decay rates), resulting in larger error bars for the unimolecular rates. Furthermore Zhou *et al.* did not investigate the temperature dependence.^19^

Diffusion would have a more significant effect at low pressures as the diffusion coefficient is inversely proportional to the pressure. However, Zhou *et al.* only argued that their experiments should be in the laminar flow condition (*i.e.*, no turbulence), without estimating the effect of diffusion.^19^ A simple but useful estimation of the diffusion effect can be found in textbooks^35^ as*z*_rms_ = (2*Dt*)^0.5^where *z*_rms_ is the root-mean-square value of the one-dimensional displacement of the molecule; *D* is the diffusion coefficient and *t* is the elapsed time. For CO_2_–O_2_ diffusion, *D* = 0.14 cm^2^ s^−1^ at 760 torr,^35^ 10.6 cm^2^ s^−1^ at 10 torr (12.7 cm^2^ s^−1^ for a more realistic estimation for their Criegee experiment at 10 torr,^19^ see ESI†). If *t* = 10 ms, we would have *z*_rms_ = 0.46 cm, which is already larger than the pump beam radius (0.3 cm) and the probe beam radius (0.1 cm) in the experiments of Zhou *et al.*^19^ This rough estimation indicates that at a low pressure of *ca.* 10 torr, the diffusion length of the molecule is quite significant even for a short period of 10 ms.

Fig. S8† shows more-realistic simulation results for cases similar to the experimental conditions of Zhou *et al.* (with orthogonal pump and probe laser beams).^19^ Again, the diffusion loss has a significant effect of about 50 s^−1^ (10 torr) to ≈< 10 s^−1^ (100 torr) (see ESI†), indicating that the low-pressure results by Zhou *et al.* should be corrected by considering the diffusion loss.

### Fate of *syn*-CH_3_CHOO in troposphere

Previous investigations^6^ have pointed out that unimolecular decay is one of the main processes of Criegee intermediates in the atmosphere. For *syn* conformers, the unimolecular decay would be the predominant process.^4^ For *syn*-CH_3_CHOO, we list the effective pseudo-first-order rates of some important processes in Table 2. It is clear that the unimolecular decomposition is the predominate decay pathway of *syn*-CH_3_CHOO in the troposphere. This fast unimolecular decay would control the steady-state concentration of *syn*-CH_3_CHOO. As a result, the probability for *syn*-CH_3_CHOO to oxidize SO_2_ would be low due to its low concentration. For other Criegee intermediates of similar structures (alkyl substitution at the *syn* position), the situation is expected to be similar. As mentioned above, the thermal decomposition rate is quite sensitive to temperature. This work gives temperature dependent value of *k*_uni_, which allows modelers to better estimate the unimolecular decay rates of *syn*-CH_3_CHOO at atmospherically relevant temperatures.

**Table 2:** Comparison of the effective first-order rate coefficients for *syn*-CH_3_CHOO at 298 K

Process	Assumed coreactant concentration/cm^−3^	Bimolecular rate coefficient/cm^3^ s^−1^	Effective first-order rate coefficient/s^−1^
Thermal decomposition	—	—	146 ± 31^a^
*syn*-CH_3_CHOO + H_2_O	3.8 × 10^17^ (50% RH)	<1 × 10^−16,^^b^	<38
*syn*-CH_3_CHOO + SO_2_	9 × 10^11^ (35 ppbv)	(2.4–2.9) × 10^−11,^^c^	22–26

a This work. Note that the value of *k*_uni_ is quite sensitive to temperature.

b Upper limit, this work.

c The values are reported by Taatjes *et al*.^12^ and Sheps *et al*.^25^

## Conflicts of interest

There are no conflicts to declare.

## Supplementary Material

RA-010-D0RA01406K-s001
