# Supplementary material for: Unimolecular decomposition rates of a methyl-substituted Criegee intermediate syn-CH3CHOO
Source: RSC Adv. 2020 Feb 28;10(14):8518–24. doi: 10.1039/d0ra01406k (PMC9049986; doi:10.1039/d0ra01406k)
Supplement: RA-010-D0RA01406K-s001 [file RA-010-D0RA01406K-s001.pdf]

Electronic Supplementary Material (ESI) for RSC Advances.
This journal is © The Royal Society of Chemistry 2020

Electronic Supplementary Information

Unimolecular Decomposition Rates of a Methyl-substituted Criegee Intermediate *syn*-CH_3CHOO

Yu-Lin Li,[1,2] *Mei-Tsan Kuo,*[1] *and Jim Jr-Min Lin**[1,2]

[1]Institute of Atomic and Molecular Sciences, Academia Sinica, Taipei 10617, Taiwan

[2]Department of Chemistry, National Taiwan University, Taipei 10617, Taiwan.

*Corresponding Author: E-mail: jimlin@gate.sinica.edu.tw

ORCID: Jim Jr-Min Lin: 0000-0002-8308-2552

Contents

Summary of experimental conditions (Tables S1-S4) S2

Fitted parameters as a function of [H_2O] (Figure S1) S6

Plot of k_{obs} as a function of [*syn*-CH_3CHOO]$_0$ at different pressures. (Figure S2) S7

Effect of H_2O reaction (Figures S3-S4) S8

Effect of second-order reactions (Figure S5) S10

Modelling of diffusion and wall loss (Tables S5-S6) S11

Simulation of diffusion loss for our experiments (Figures S6-S7) S13

Simulation of diffusion loss for the experiments of Zhou *et al.* (Figure S8) S15

Error estimation (Table S7) S17

Representative time traces for the thermal decomposition of *syn*-CH_3CHOO (Figures S9-S28) S18

Representative time traces for the reaction of CH3CHOO and water vapor (Figures S29-S32) S38

References S42

Table S1. Summary of experimental conditions for the reaction of *syn*-$CH_3CHOO + H_2O$

Exp. #	Temp. / K	Laser fluence / mJ cm^{-2}	$[CH_3CHI_2]$ / 10^{13} cm^{-3}	P_{O2} / Torr	P_{total} / Torr	k_w / 10^{-17} cm^3s^{-1}	# of data points
W1-1	318.5	1.8	8.4	10.1	300.1	16.9 ± 3.4[a]	12
W1-2	318.5	1.8	5.1	10.2	300.5	16.4 ± 8.1	12
W1-3	318.5	1.8	11.6	10.1	300.2	23.6 ± 6.0	12
W1-4	318.5	1.8	8.4	10.1	300.9	24.4 ± 3.4	14
W2-1	308.4	1.7	7.9	10.3	301.4	13.8 ± 2.4	14
W2-2	308.4	1.7	5.2	10.3	305.1	8.5 ± 5.0	12
W2-3	308.3	1.7	13.6	10.3	305.0	11.5 ± 2.4	12
W2-4	308.3	1.7	7.8	10.3	305.1	9.7 ± 3.7	8
W3-1	298.8	1.6	8.1	10.1	301.5	5.7 ± 2.4	8
W4-1	297.0	1.7	7.4	10.3	303.2	16.7 ± 2.0	10
W4-2	297.3	1.6	12.5	10.5	104.5	13.7 ± 1.6	10
W4-3	297.4	1.6	17.9	10.2	702.0	17.9 ± 2.4	10

[a] Error bar is one standard deviation obtained from the linear fitting of k_{obs} against $[H_2O]$.

Table S2. Summary of experimental conditions for *syn*-CH_3CHOO thermal decomposition at various temperatures. P_{O2} = 10.0~10.3 Torr; P_{total} = 300~302 Torr.

Exp. #	Temp. / K	Laser fluence / mJ cm^{-2}	[H_2O] /10^{17} cm^{-3}	k_{uni} (assuming k_w=0) / s^{-1}	k_{uni} / s^{-1}	# of data points
1-1	298.7	1.7	3.50	193.2 ± 34.9	162.2 ± 34.9	6
1-2	298.6	3.0	3.41	175.2 ± 34.0	145.3 ± 34.0	6
1-3	298.6	1.0	3.34	174.1 ± 33.5	144.7 ± 33.5	6
1-4	298.7	1.0	3.27	163.7 ± 33.1	134.8 ± 33.1	6
1-5	298.7	3.0	3.33	179.0 ± 33.5	149.6 ± 33.5	6
1-6	298.7	1.7	3.36	180.3 ± 33.7	150.6 ± 33.7	6
2-1	298.7	1.7	1.94	164.5 ± 23.5	147.3 ± 23.5	16
2-2	308.9	1.6	3.01	257.4 ± 44.6	218.6 ± 44.6	16
2-3	308.9	1.6	3.68	257.4 ± 52.3	210.0 ± 52.3	16
2-4	318.7	1.6	5.28	402.6 ± 106.4	306.7 ± 106.4	16
2-5	318.7	1.6	3.61	352.2 ± 80.2	286.6 ± 80.2	17
3-1	298.8	1.7	1.94	160.8 ± 23.5	143.6 ± 23.5	12
3-2	288.3	1.6	0.66	105.2 ± 14.4	101.3 ± 14.4	16
3-3	288.3	1.6	1.27	96.9 ± 15.8	89.5 ± 15.8	16
3-4	288.3	1.6	1.60	105.5 ± 16.7	96.2 ± 16.7	16
4-1	287.8	1.7	1.92	103.7 ± 17.7	92.7 ± 17.7	8
4-2	287.8	1.7	3.12	123.8 ± 22.6	106.0 ± 22.6	8
4-3	298.3	1.7	2.49	180.0 ± 27.0	158.4 ± 27.0	8
4-4	298.4	1.7	4.41	191.0 ± 41.6	152.6 ± 41.6	8
4-5	308.4	1.7	1.92	225.6 ± 32.8	201.3 ± 32.8	8
4-6	308.4	1.7	4.42	284.8 ± 60.2	228.8 ± 60.2	8
4-7	318.5	1.7	2.32	308.1 ± 62.3	266.2 ± 62.3	8
4-8	318.4	1.6	3.34	351.2 ± 75.9	290.9 ± 75.9	8
4-9	288.1	1.6	2.43	116.0 ± 19.7	102.0 ± 19.7	8
4-10	288.0	1.6	1.92	121.7 ± 17.8	110.6 ± 17.8	8
5-1	278.1	1.7	0.61	66.9 ± 15.1	64.6 ± 15.1	8
5-2	278.1	1.7	0.85	57.3 ± 15.2	54.2 ± 15.2	8
5-3	278.0	1.7	1.23	67.3 ± 15.6	62.7 ± 15.6	8
5-4	278.0	1.7	1.76	90.2 ± 16.3	83.6 ± 16.3	8
5-5	278.0	1.7	0.83	75.0 ± 15.2	71.9 ± 15.2	8

[a] Error bar is one standard deviation, which will be discussed in Error Estimation.

Table S3. Summary of experimental conditions for *syn*-CH_3CHOO thermal decomposition at various pressures. P_{O2} = 10.0~10.6 Torr; Temp.= 296.8~298.7 K.

Exp. #	Laser fluence / mJ cm^{-2}	[H_2O] /10^{17} cm^{-3}	P_{total} / Torr	k_{uni} (assuming k_w=0) / s^{-1}	k_{uni} / s^{-1}	# of data points
P1-1	1.7	1.50	101.2	127.8 ± 14.4	117.2 ± 14.4[a]	8
P1-2	1.7	2.54	101.1	139.4 ± 20.4	121.5 ± 20.4	8
P1-3	1.6	3.58	101.4	145.2 ± 27.0	120.1 ± 27.0	8
P1-4	1.6	3.05	301.7	171.6 ± 28.0	145.3 ± 28.0	8
P1-5	1.6	1.43	101.1	138.7 ± 14.1	128.7 ± 14.1	8
P2-1	1.7	1.48	100.9	131.4 ± 14.3	121.0 ± 14.3	8
P2-2	1.7	2.57	101.5	134.5 ± 20.6	116.4 ± 20.6	8
P2-3	1.6	3.27	502.3	199.0 ± 33.7	169.9 ± 33.7	16
P2-4	1.6	3.45	702.5	210.8 ± 36.0	179.0 ± 36.0	7
P2-5	1.6	2.54	100.5	138.7 ± 21.8	120.8 ± 21.8	8
P3-1	1.7	3.39	502.9	182.1 ± 32.7	151.9 ± 32.7	8
P3-2	1.7	3.46	702.0	240.4 ± 34.3	208.5 ± 34.3	8
P3-3	1.7	3.04	299.8	160.8 ± 30.5	134.7 ± 30.5	8
P3-4	1.6	3.44	697.8	210.3 ± 35.4	178.6 ± 35.4	8
P3-5	1.6	3.30	498.9	201.9 ± 33.3	172.6 ± 33.3	8
P4-1	1.7	2.87	303.1	158.5 ± 29.3	133.8 ± 29.3	8
P4-2	1.6	2.43	104.4	130.0 ± 23.2	112.9 ± 23.2	8
P4-3	1.6	3.30	701.0	199.8 ± 34.3	169.4 ± 34.3	8

[a] Error bar is one standard deviation, which will be discussed in Error Estimation.

Table S4. Summary of experimental conditions for wall loss estimation

Exp. #	Temp. / K	Laser fluence / mJ cm^{-2}	P_{O2} / Torr	P_{total} / Torr	$k_{intercept}$ / s^{-1}	# of data points
L1	279.1	1.1	10.1	301.9	6.6 ± 1.8[a]	12
L2	278.9	1.0	10.0	299.1	6.7 ± 3.6	12
L3	289.0	1.0	10.1	302.0	1.2 ± 1.8	12
L4	299.6	1.0	10.1	302.0	1.0 ± 2.0	12
L5	298.3	3.1	10.4	303.9	7.7 ± 1.4	7
L6	298.2	2.4	10.4	304.1	6.0 ± 2.3	7
L7	298.2	1.7	10.4	304.4	5.3 ± 2.2	7
L8	299.1	1.0	10.6	303.7	6.7 ± 1.3	10
L9	299.0	1.0	10.6	303.9	6.6 ± 1.5	10
L10	309.4	1.0	10.1	299.9	6.4 ± 1.0	12
L11	320.1	1.0	10.1	299.5	9.5 ± 2.1	12
L12	323.8	1.6	11.0	316.8	10.1 ± 0.9	12
L13	323.9	1.6	11.0	317.1	9.2 ± 1.0	12
L14	346.4	1.6	11.3	324.3	11.9 ± 1.8	11
L15	344.1	1.6	11.3	324.4	8.6 ± 2.8	11
L16	297.8	1.0	10.2	102.7	6.9 ± 1.9	11
L17	297.7	1.0	10.3	305.2	3.9 ± 1.7	11
L18	298.0	1.0	10.2	509.4	8.2 ± 2.2	12
L19	298.2	1.0	10.0	695.4	9.5 ± 4.4	12

[a] Error bar is one standard deviation obtained from the linear fitting of k_{obs} against $[CH_2OO]_0$.

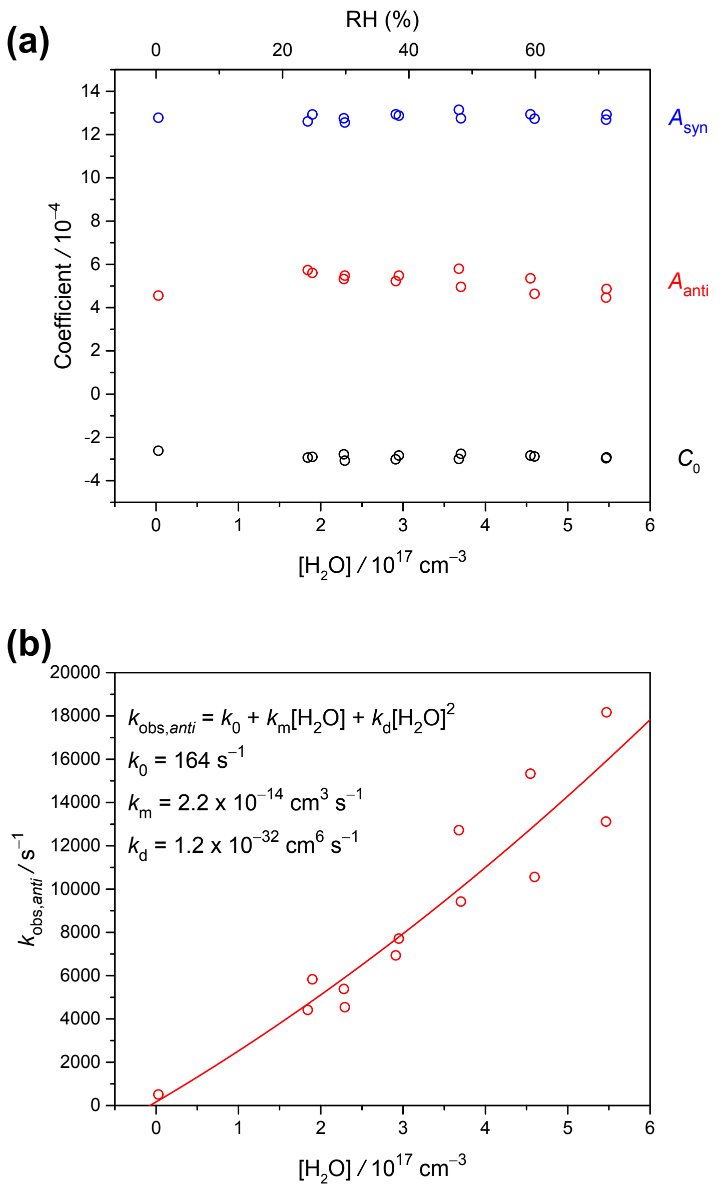

Figure S1. Fitted parameters of the double exponential model (Eq 1) for Exp. W2-3 at 308 K. (a) Pre-exponential factors and the offset plotted as functions of [H_2O]. (b) The observed decay rate coefficient of *anti*-CH_3CHOO ($k_{obs,anti}$) as a function of [H_2O].

It is not easy to fit the signal trace at [H_2O] = 0 because the difference of τ_{anti} and τ_{syn} are not significant. After fitting all signal traces at [H_2O] > 0, we extrapolated the fitted parameters (A_{anti} A_{syn}, τ_{anti} and τ_{syn}) to their values at [H_2O] = 0 and use the extrapolated values as the initial values to perform the fitting for the case of [H_2O] = 0.

As shown in **Figure S1**, the signal amplitudes (A_{anti}, A_{syn}) and the offset (C_0) are independent on [H_2O] (**Figure S1** (a)), which reflects the stability of the double exponential fitting. The large dispersion of $k_{obs,anti}$ at high [H_2O] (**Figure S1** (b)) is due to the small contribution of *anti*-CH_3CHOO absorption at 340 nm (smaller signal compared to that of *syn*-CH_3CHOO).

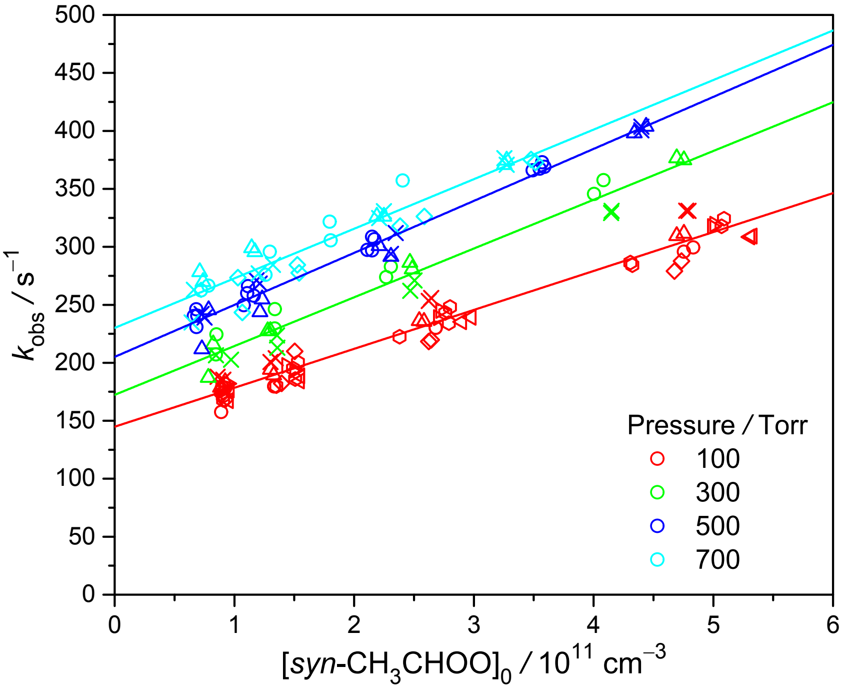

Figure S2. Plot of k_{obs} as a function of [*syn*-$CH_3CHOO]_0$ at different pressures. The experiments were conducted at 298 K and detailed experimental conditions are shown in **Table S3**.

Effect of H_2O reaction

(i) Temperature effect

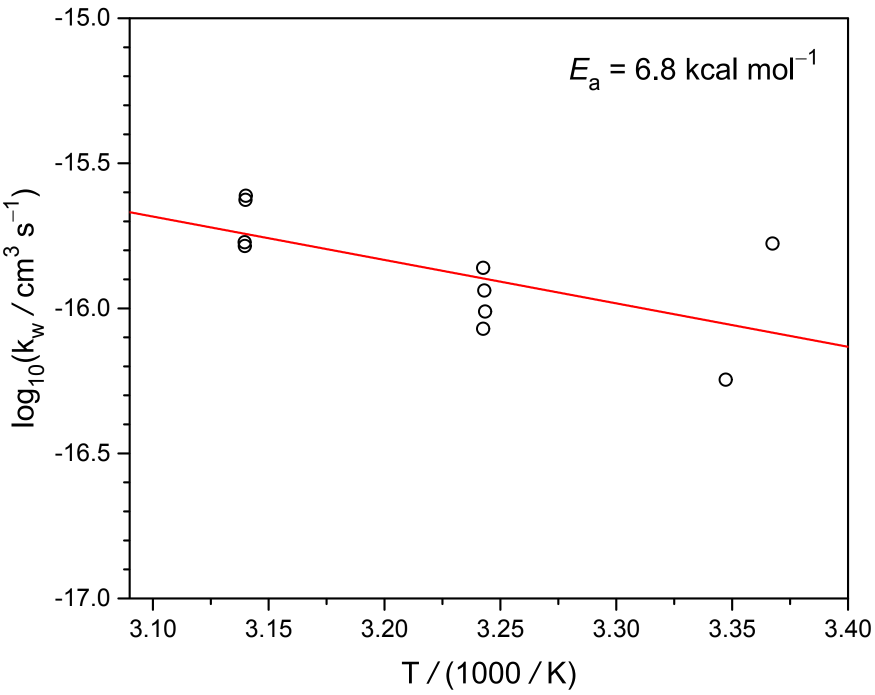

Figure S3. Arrhenius plot of the rate coefficient of *syn*-CH_3CHOO reaction with water vapor. The experimental conditions are shown in **Table S1**.

(ii) Pressure effect

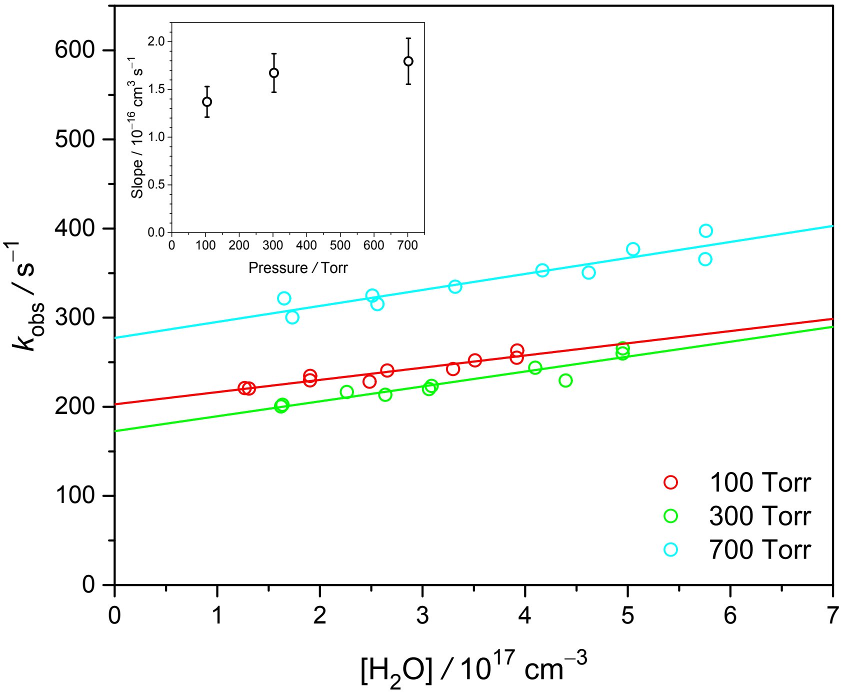

Figure S4. Plot of k_{obs} as a function of [H_2O] at three pressures under 297 K (Exp. W4-1 to W4-3, see **Table S1** for details). The lines are linear fit to the data. **Inset**: The plot of slopes versus pressure. Error bar is one standard deviation obtained from the linear fitting of k_{obs} against [H_2O].

We found the reaction of *syn*-CH_3CHOO with water vapor has a weak pressure dependence for 100 to 700 Torr. To ensure the consistency of $k_w(P,T)$, we normalized the values of k_w obtained from Figure S4 to be consistent with the value obtained from the Arrhenius plot at 298 K and 300 Torr (Figure S3). For k_w at 500 Torr, we used the average value of k_w at 300 and 700 Torr.

Effect of second-order reactions

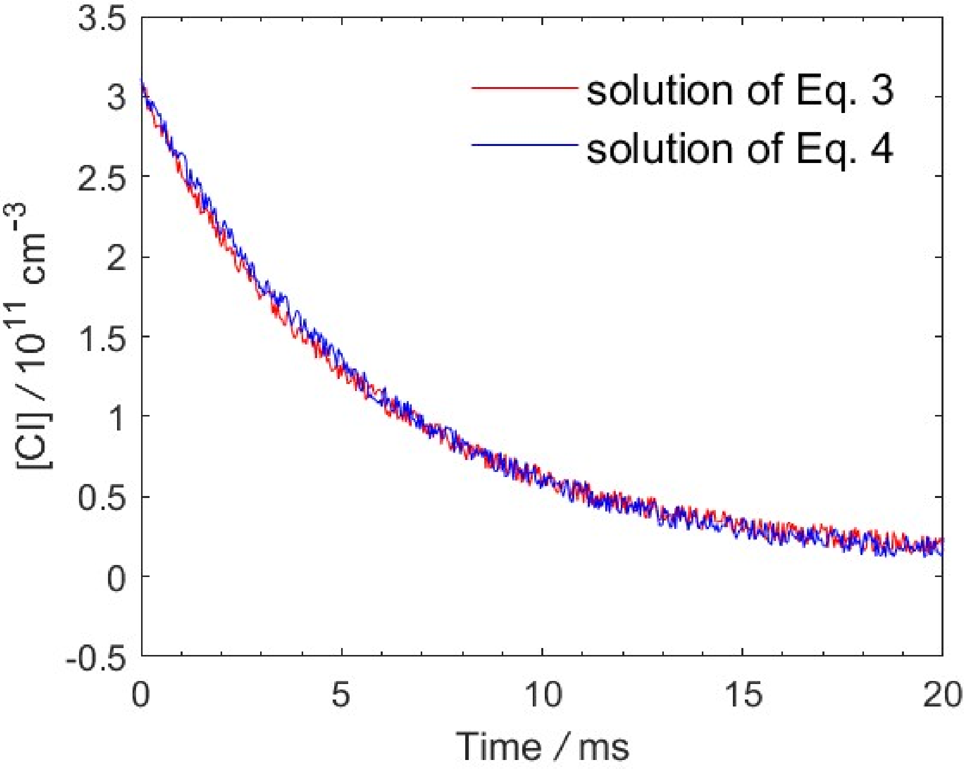

Figure S5. Simulated time traces of the solutions of (Eq 3) (red) and (Eq 4) (blue) for $[CI]_0 = 3x10^{11}$ cm^{-3}, $k_1 = 150\ s^{-1}$, $k_2 = 1.6x10^{-10}\ cm^3s^{-1}$. Scaled random numbers have been added to the solutions to mimic the noise.

The solution of (Eq 3): $$[\mathrm{CI}]_t = [\mathrm{CI}]_0 \frac{\left(1+\frac{k_2[\mathrm{CI}]_0}{k_1}\right)^{-1} \mathrm{e}^{-k_1 t}}{1-\left(1+\frac{k_1}{k_2[\mathrm{CI}]_0}\right)^{-1} \mathrm{e}^{-k_1 t}}$$

The solution of (Eq 4): $$[\mathrm{CI}]_t \cong [\mathrm{CI}]_0 \mathrm{e}^{-(k_1+\frac{1}{2}k_2[\mathrm{CI}]_0)t}$$

Modelling of diffusion and wall loss

(i) Estimation of the diffusion coefficient of CI

The diffusion coefficient of CI, which has not been reported, is estimated with the approach proposed by Fuller et al.:[1]

$$D_{\text{CI-A}} = \frac{1\times10^{-3} T^{1.75} (1/\mathrm{M}_{\text{CI}} + 1/\mathrm{M}_{\text{A}})^{0.5}}{P(\Sigma_{\text{CI}}^{1/3} + \Sigma_{\text{A}}^{1/3})^2}$$

Where $D_{\text{CI-A}}$ is the binary diffusion coefficient in cm^2/s, T is the temperature in K, P is the pressure in atm, and M_{CI} and M_{A} is the molar mass of CI and species A, respectively. The dimensionless diffusion volume, Σ, of various gases have been reported elsewhere, whereas that of CI is estimated by summing up the contributions of every atoms of CI.[1] Since there are only trace amounts of CI in the system, the diffusion constant of CI in a homogeneous gas mixture is estimated with the Blanc's law:[2]

$$D_{\text{CI-mix}} = \left(\frac{\chi_{\text{A}}}{D_{\text{CI-A}}} + \frac{\chi_{\text{B}}}{D_{\text{CI-B}}}\right)^{-1}$$

χ_{A} and χ_{B} are the mole fractions of gas species A and B, and $D_{\text{CI-A}}$ and $D_{\text{CI-B}}$ are the diffusion constants of CI in gases A and B, respectively.

The diffusion volumes of various gases and CI, and the diffusion constants of various reaction conditions are summarized in Tables S5 and S6.

Table S5. The molecule weight and diffusion volume of various species

Species	Molecule weight /amu	Diffusion Volume[1]
N_2	28.00	17.9
O_2	31.99	16.6
Ar	39.95	16.1
CH_2OO	45.98	31.42[a]
CH_3CHOO	59.98	51.88[a]

[a] Diffusion volume is calculated by summing up the contributions of the atoms.[1]

Table S6. Experimental condition for the simulation and the calculated diffusion coefficient of various Criegee intermediate (CI).

Sim#	CI	Buffer Gas	T /K	P_{O2} /Torr	P_{Buff} /Torr	$D_{CI\text{-}mix}$ /cm^2s^{-1}
1	CH_2OO	N_2	298	10	90	1.40
2	CH_2OO	N_2	298	10	290	0.41
3	CH_2OO	N_2	298	10	490	0.24
4	CH_2OO	N_2	298	10	690	0.17
5	CH_3CHOO	Ar	298	2.11	7.89	12.74
6	CH_3CHOO	Ar	298	4.48	20.52	4.81
7	CH_3CHOO	Ar	298	5.13	44.87	2.08
8	CH_3CHOO	Ar	298	5.39	69.61	1.31
9	CH_3CHOO	Ar	298	5.53	94.47	0.95

(ii) Simulation of the diffusion loss

The diffusion and wall loss of CI is estimated with the approximation of Fick's law. Considering the cylindrical symmetry, the governing equation can be simplified as:[3]

$$\frac{\partial C}{\partial t} = \frac{1}{r}\frac{\partial}{\partial r}(rD_{\text{CI-mix}}\frac{\partial C}{\partial r})$$

Where C is the concentration of targeted molecules, r is the radius, and $D_{\text{CI-mix}}$ is the diffusion coefficient of CI in the gas mixture. The governing equation of diffusion was solved with the build-in partial differential equation solver of MATLAB to obtained the time dependent concentration distribution in radial direction of the targeted molecule, $C(r,t)$.

Simulation of diffusion loss for our experiments

The inner diameter of our reaction cell is 1.9 cm. Assuming that the wall reaction is very fast that CI would be eliminated upon collision with the wall, the boundary condition can be set as:

$$C(0.95,t) = 0\ ,\ \frac{\partial C}{\partial r}(0,t)\ =\ 0$$

The second equation is to ensure the continuity of $C(r,t)$ at $r = 0$.

The effective beam size of the photolysis laser is 1.8 cm. We assume the initial density of CI is uniform within the photolysis volume and hence the initial condition is $C(r,0) = H(0.9 - r)$, where H is the Heaviside step function that $H(x)=\{0 \text{ if } x<0,\ 0.5 \text{ if } x=0,\ 1 \text{ if } x>0\}$. To simulate the observed decadence resulting from diffusion and wall lose, the size of the probe beam should also be considered. The probe beam applied in our system, which is roughly in the shape of converging cylindrical cones, is collinear to the photolysis beam in the axial direction of the reaction cell. In the simulation, we assume that the effective diameter of the probe beam is 1.3 cm. The calculated $C(r,t)$ is then weighted with the differential area at different radius, and normalized with respect to $C(t=0)$ to simulate the observed decay of absorbance. The simulated traces at various reaction conditions are shown in **Figure S7**. The maximum simulated diffusion loss rate, which occurs at the lowest pressure in our experiments (100 Torr), is less than 7 s^{-1}. This value is smaller than the observed k_{wall} obtained from the experiment of CH_2OO. Hence, we think most of the wall loss in our experiment comes from turbulence, which is more severe at higher pressures, and this assumption is consistent with the experimental observation. However, the variation of k_{wall} at different pressure and temperature is very small (**Figure S6**). Hence we reported the averaged value of k_{wall} for the further calculation of k_{uni} of *syn*-CH_3CHOO. Note that turbulence should not depend on the species of Criegee intermediates in the highly diluted experimental conditions.

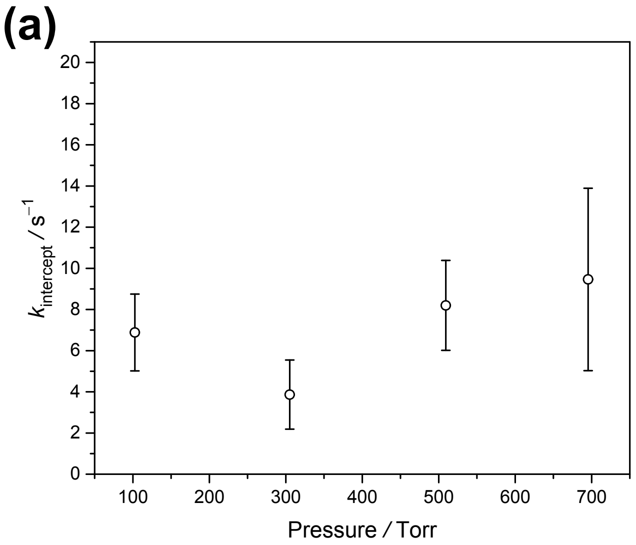

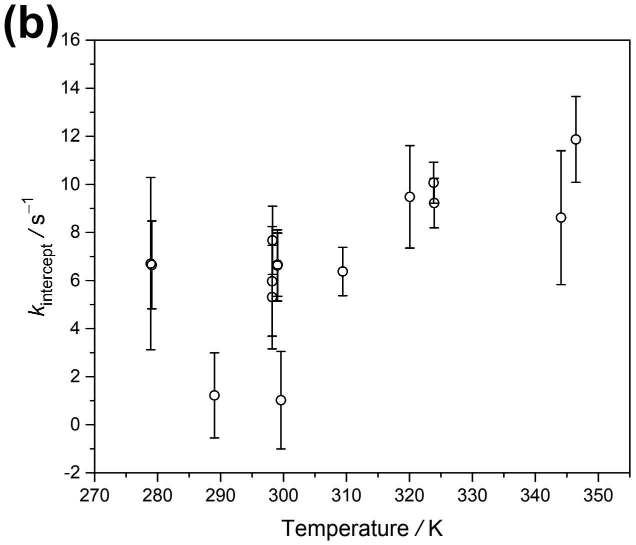

Figure S6. (a) $k_{intercept}$ as a function of pressure at 298 K (Exp. L16 to L19). (b) $k_{intercept}$ as a function of temperature at 300 Torr (Exp. L1 to L15).

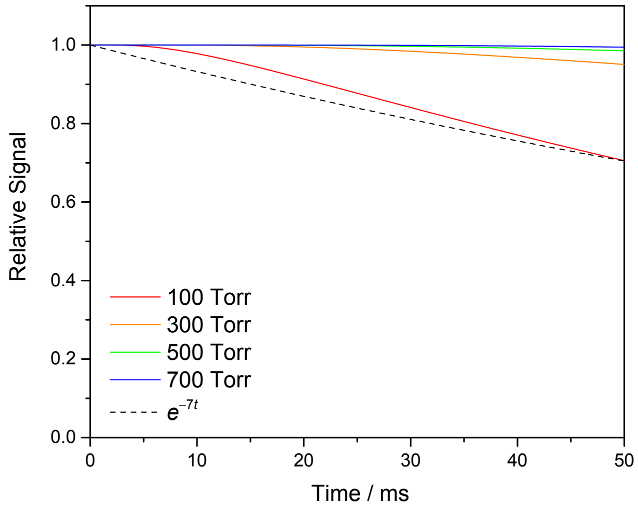

Figure S7. The simulated diffusion loss of CH_2OO for our experimental conditions. The reaction conditions are described in **Table S6** (Sim# 1-4).

Simulation of diffusion loss for the experiments of Zhou *et al.*

The experimental setup has been mentioned in their previous publication.[4] The inner diameter of the reaction cell is 5.30 cm, whereas the diameter of the photolysis laser beam is 0.6 cm. Hence, the boundary condition is set as:

$$C(2.65,t)=0\ ,\frac{\partial C}{\partial r}(0,t)\ =\ 0$$

and the initial condition is set as:

$$C(r,0)=H(0.3-r)\,.$$

The probe beam, whose diameter 0.4 cm, is perpendicular to the photolysis laser. Hence, the calculated $C(r,t)$ is weighted with the differential area at different photolysis beam radius and at different probe beam radius. The simulated decay curve (**Figure S7**) shows that the diffusion loss is quite significant. The main reasons are (i) their photolysis beam size is much smaller than that of ours and (ii) their pressures are lower. This result indicates that diffusion loss is not negligible under the experiment conditions of Zhou *et al*.

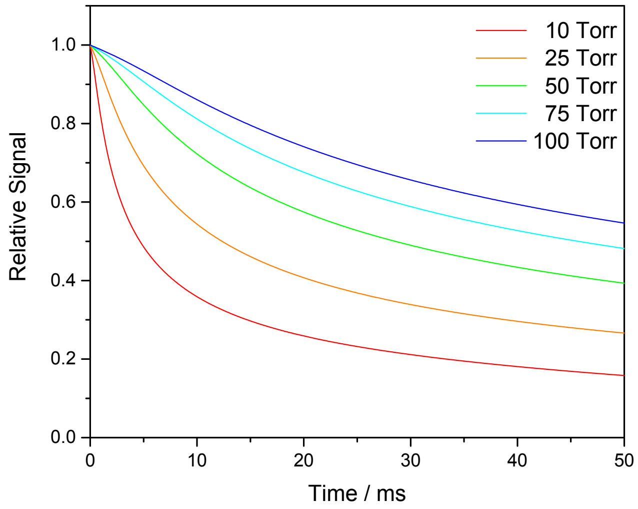

Figure S8. The simulated diffusion loss for the experiment conditions of Zhou *et al.* The reaction condition is described in **Table S6** (Sim 5-9).

Error Estimation

The thermal decomposition rate coefficient (and its error bar) is obtained by using the following formula:

$$k_{\text{uni}} = k_{\text{intercept}} - k_{\text{w}}[\text{H}_2\text{O}] - k_{\text{wall}}$$

$$\sigma_{\text{total}} = \sqrt{\sigma_{\text{intercept}}^2 + \sigma_{\text{water}}^2 + \sigma_{\text{wall}}^2}$$

(1) $\sigma_{\text{intercept}}$.

We use the standard deviation of k_{obs} at the lowest [*syn*-CH_3CHOO]$_0$ to represent the errors of the intercept rates.

(2) σ_{water}.

Due to the slow rates of the water vapor reactions, we are uncertain about $k_{\text{w}}[H_2O]$ and treat its values as the errors ($\sigma_{\text{water}} = k_{\text{w}}[H_2O]$). The rate coefficients $k_{\text{w}}(T)$ at each temperature are obtained by the Arrhenius plots (**Figure S3)**. This part preponderates at higher temperatures since this reaction has a positive temperature effect.

(3) σ_{wall}.

Because the wall loss rate has no significant temperature or pressure dependence, we can take the standard deviation of the data as its error. This term is minor.

Table S7 shows one example of the estimated error bars. The most significant term is the water reaction term, but this term depends strongly on temperature. The reported value and error of k_{uni} at each temperature are the average numbers.

Table S7. Example of the estimated errors

Exp. # 1-2 (298 K)	Absolute error of each term / s^{-1}		
k_{uni} / s^{-1}	Intercept ($\sigma_{\text{intercept}}$)	Water reaction (σ_{water})	Wall loss (σ_{wall})
145 ± 34	15	30	5.7

Representative time traces for the thermal decomposition of *syn*-CH_3CHOO

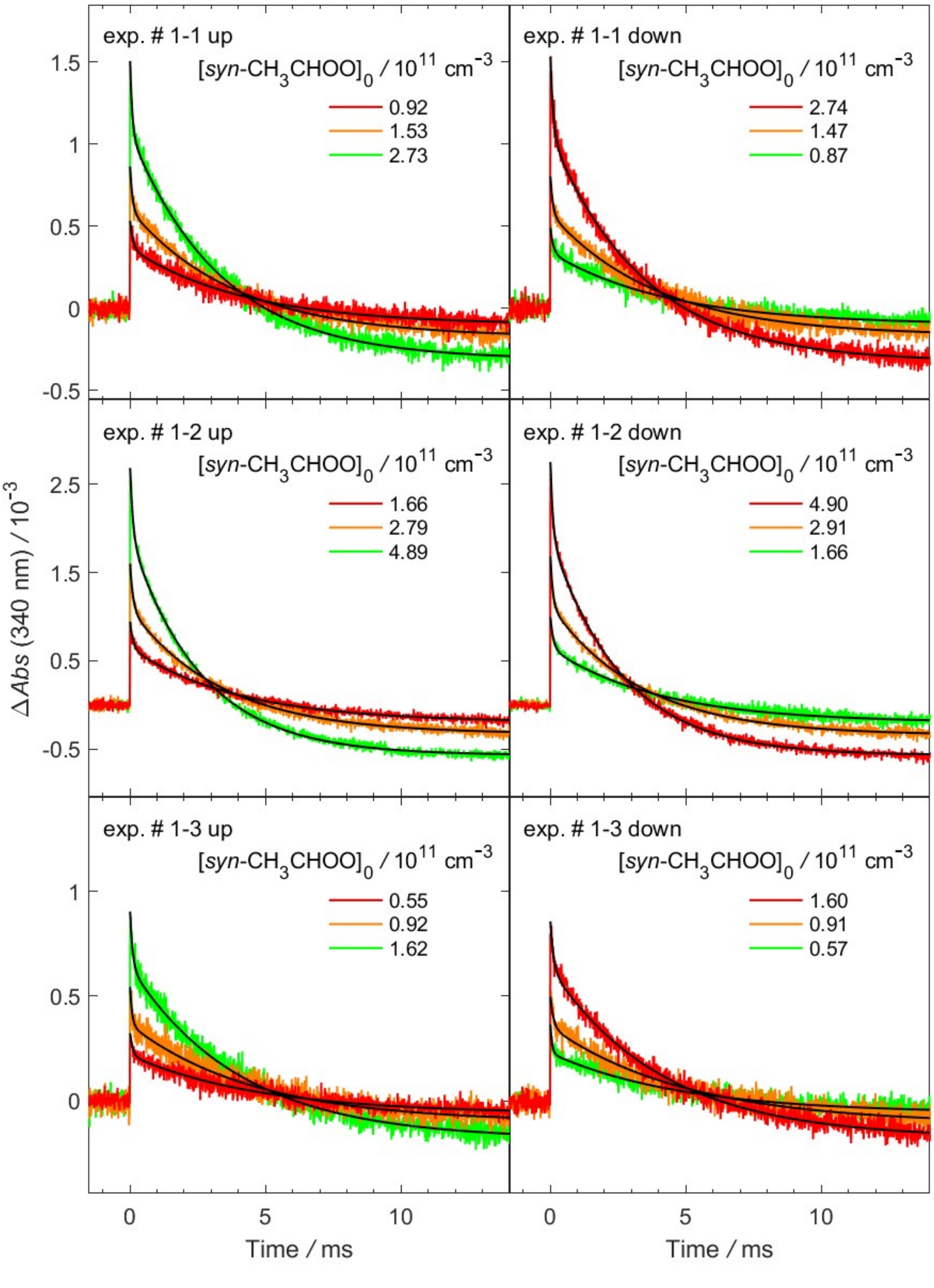

Figure S9. Representative time traces of CH_3CHOO at 340±5 nm under different [*syn*-CH_3CHOO]$_0$ (exp. # 1-1 to 1-3). [*syn*-CH_3CHOO]$_0$ was estimated by using the Beer-Lambert law with fitted peak height absorbance, the cross section $\sigma_{syn} = 1.19 \times 10^{-17}$ cm^{-2} at 340 nm[5] and effective length L = 426 cm. The photolysis laser pulse sets the time zero. In each experiment, [*syn*-CH_3CHOO]$_0$ was scanned from the minimum to the maximum (labeled as "up") and from the maximum to the minimum (labeled as "down"). For each trace, the black line is the two-exponential fit to the signal of CH_3CHOO. The negative baseline was due to the depletion of the precursor CH_3CHI_2.

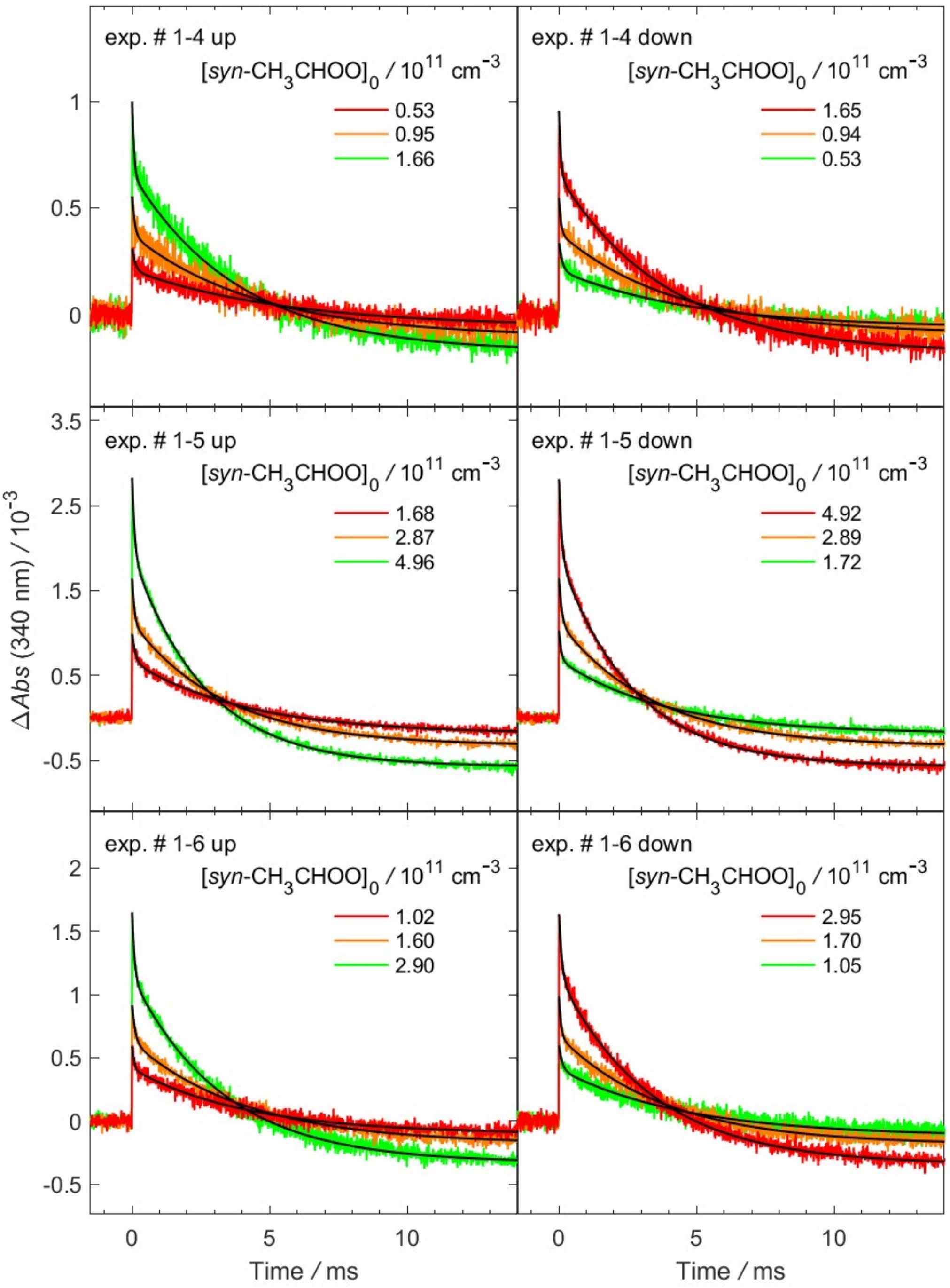

Figure S10. As Figure S9, but for different experiment sets (exp. # 1-4 to 1-6).

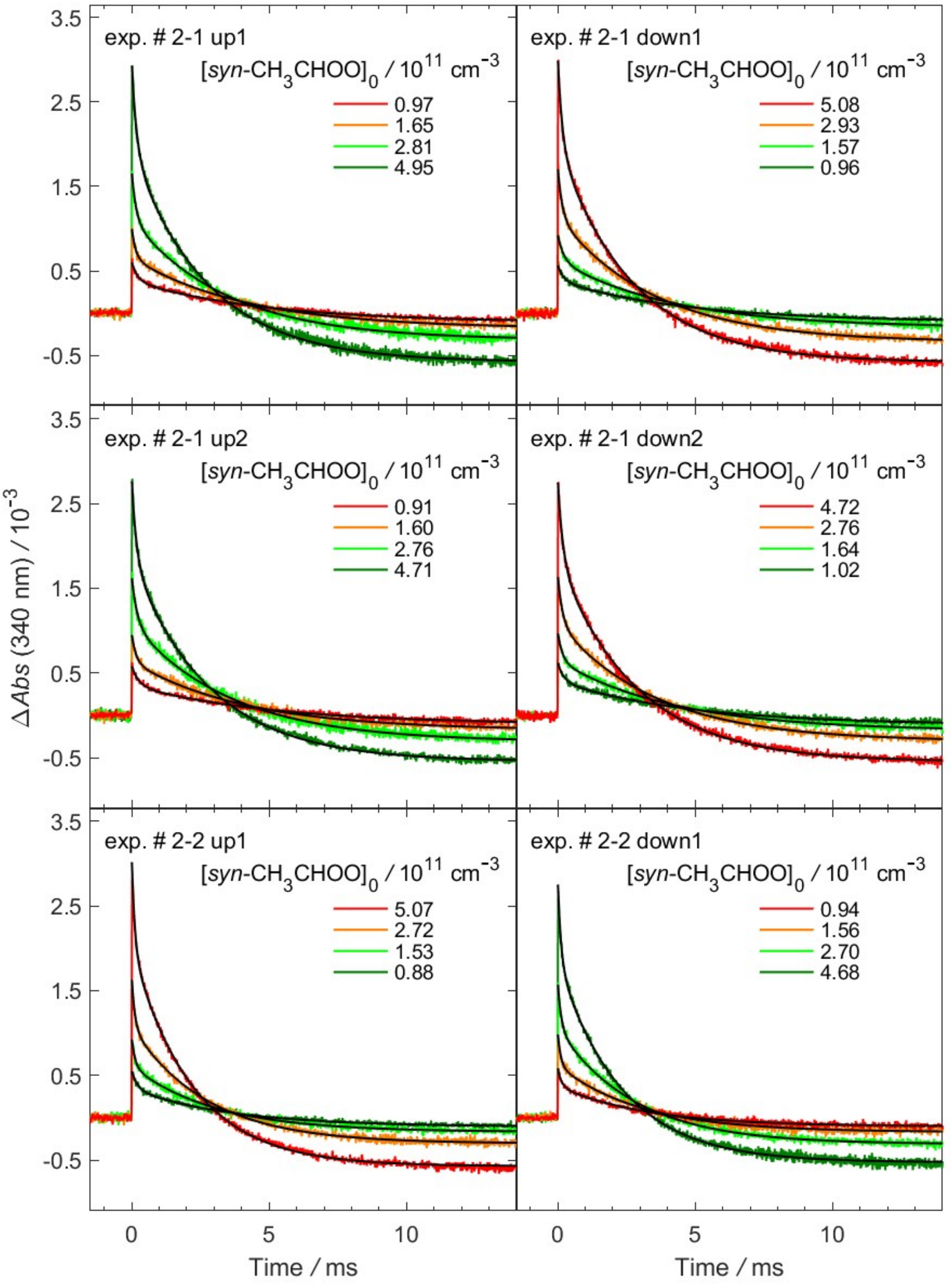

Figure S11. As Figure S9, but for different experiment sets (exp. # 2-1 to 2-2). For some Exp sets, we scanned two round trips for [*syn*-CH_3CHOO]$_0$ with the scan sequence of "up 1", "down 1", "up 2", then "down 2".

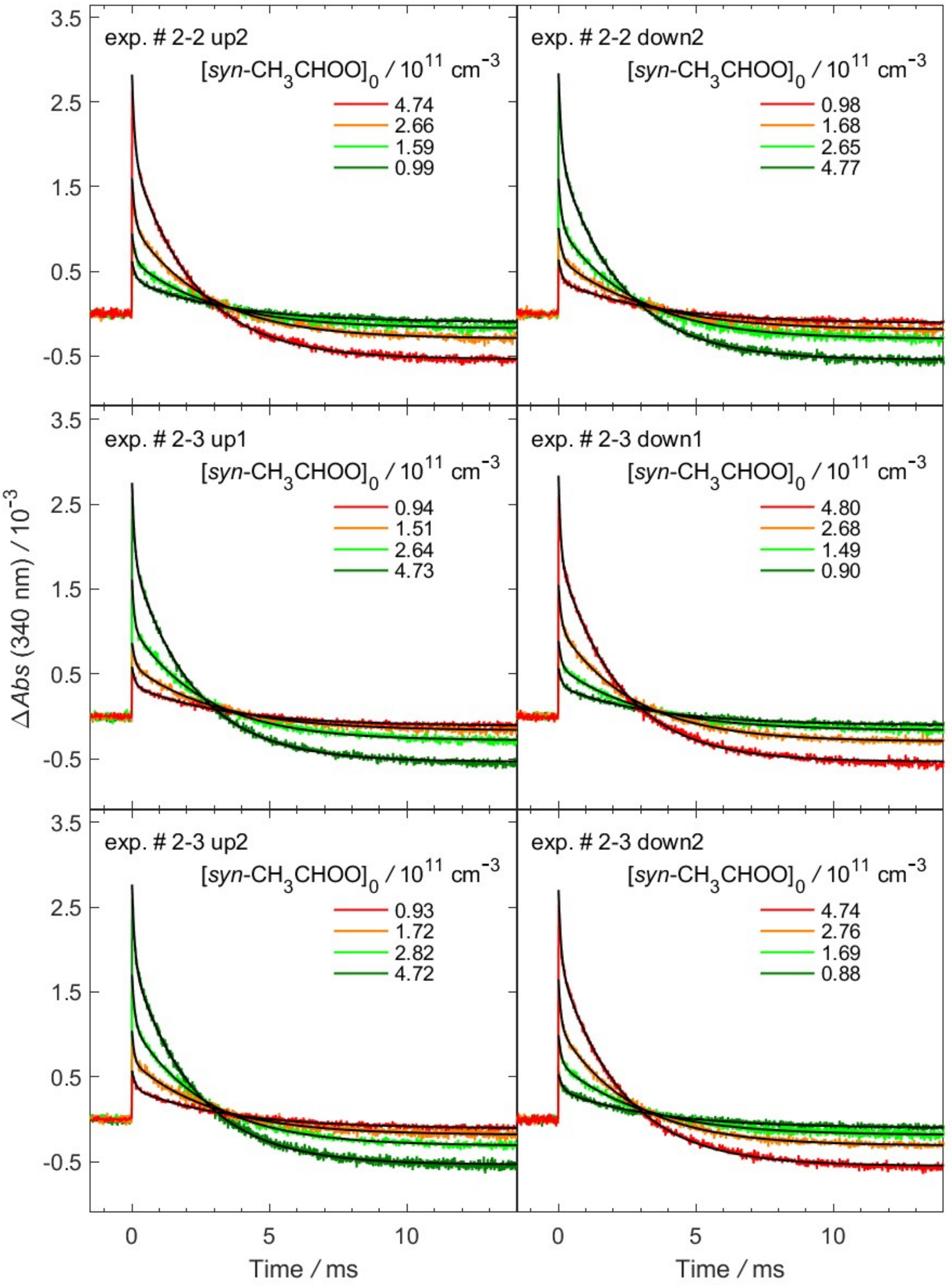

Figure S12. As Figure S9, but for different experiment sets (exp. # 2-2 to 2-3).

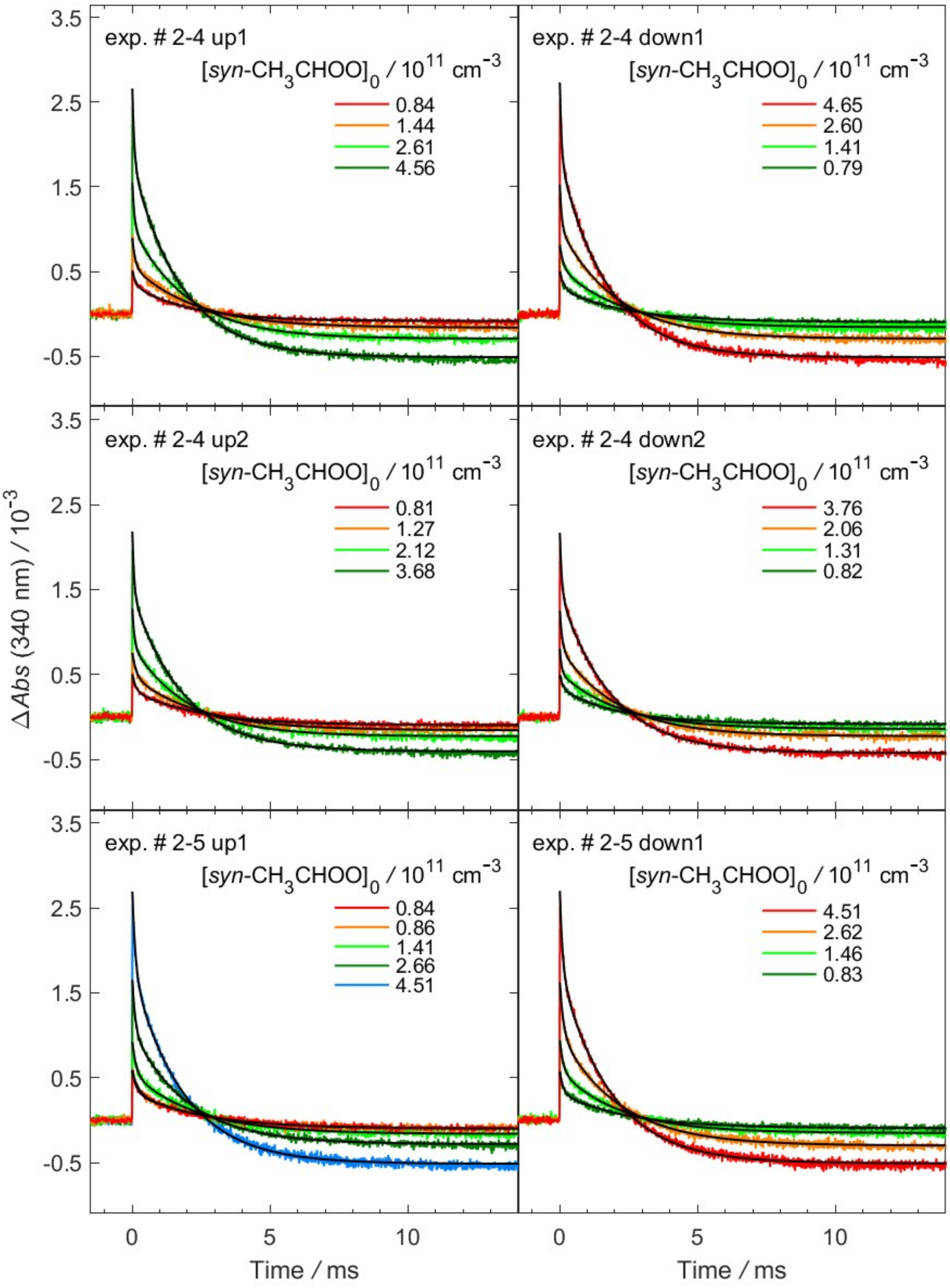

Figure S13. As Figure S9, but for different experiment sets (exp. # 2-4 to 2-5).

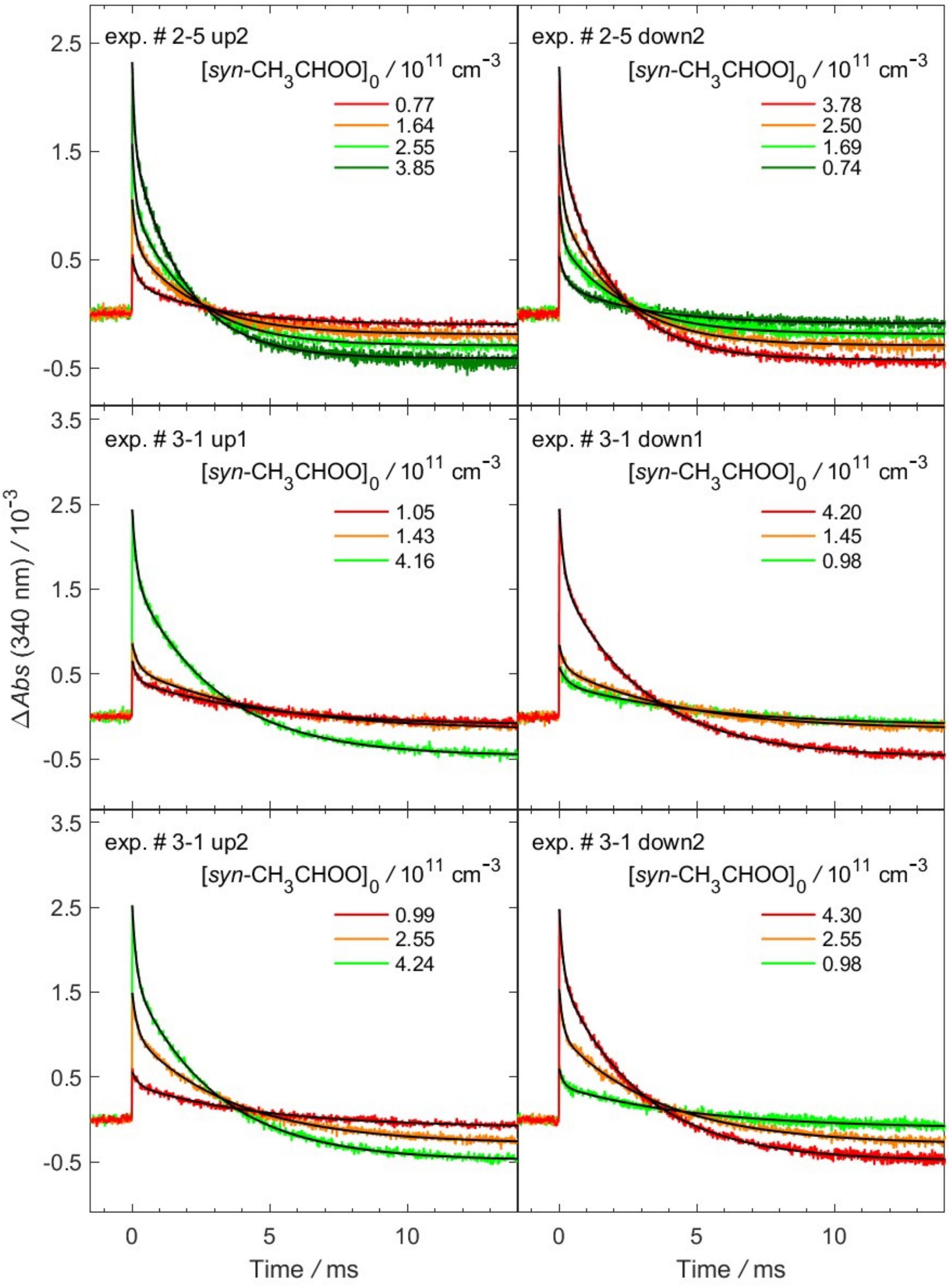

Figure S14. As Figure S9, but for different experiment sets (exp. # 2-5 to 3-1).

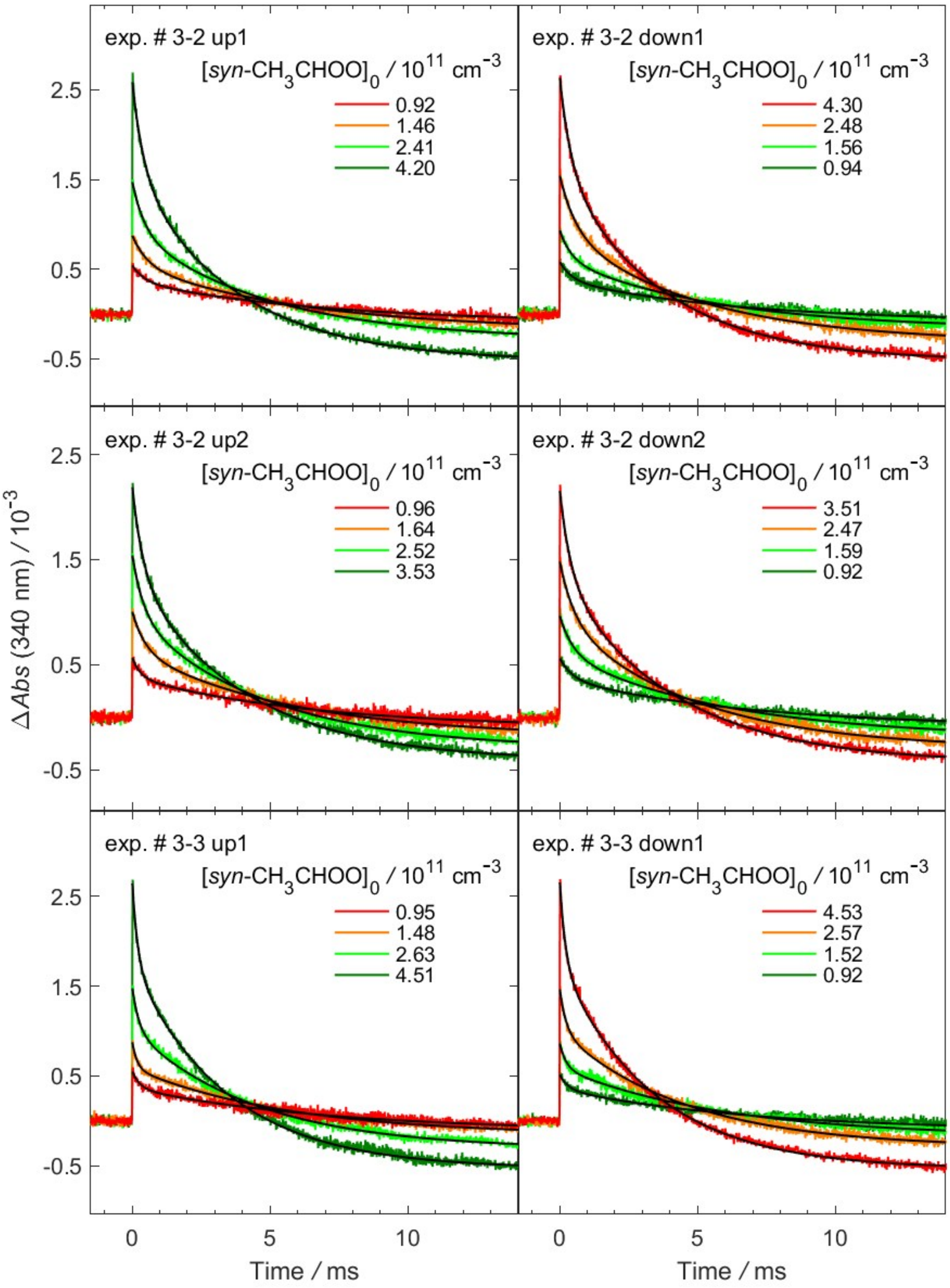

Figure S15. As Figure S9, but for different experiment sets (exp. # 3-2 to 3-3).

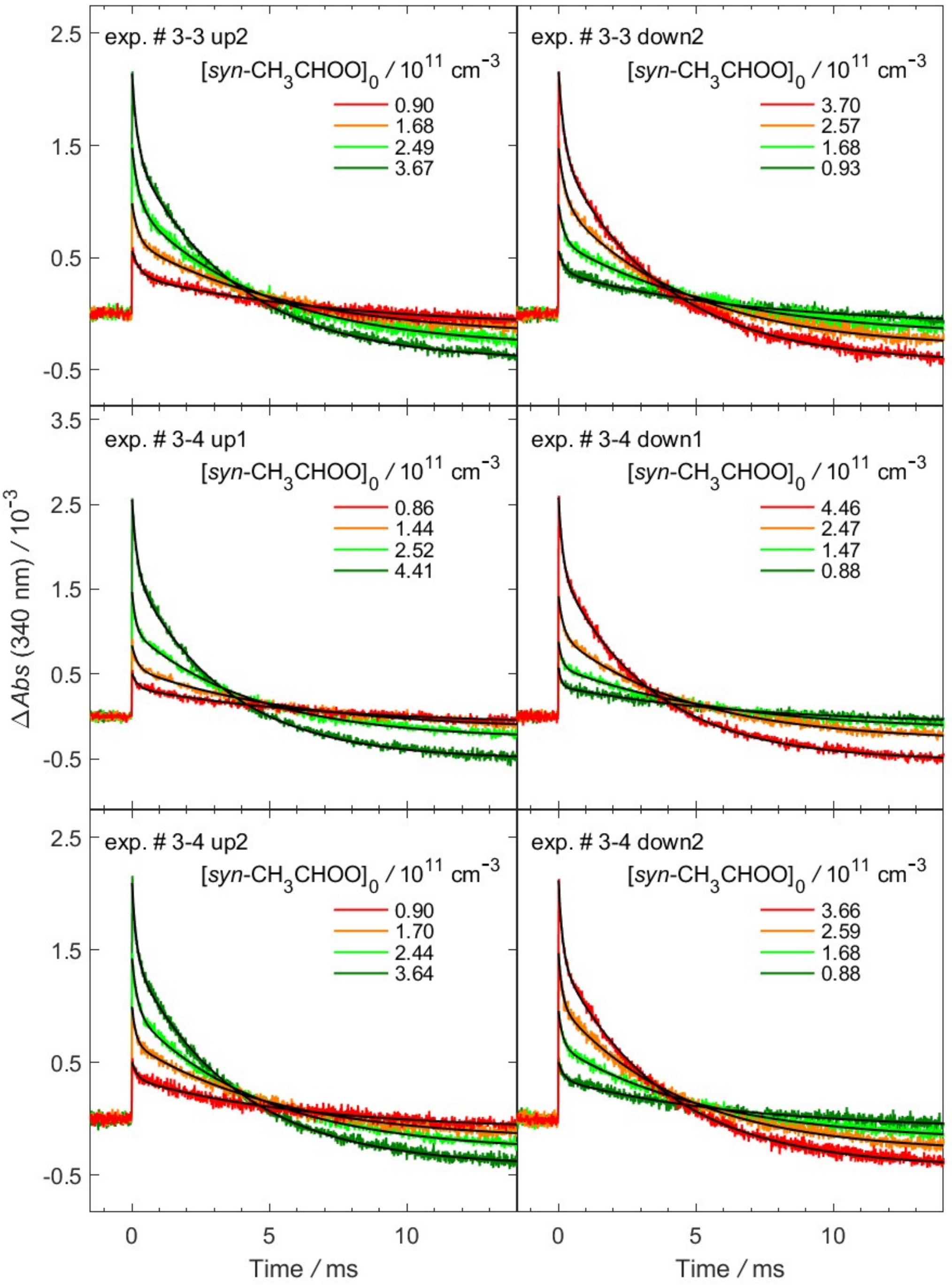

Figure S16. As Figure S9, but for different experiment sets (exp. # 3-3 to 3-4).

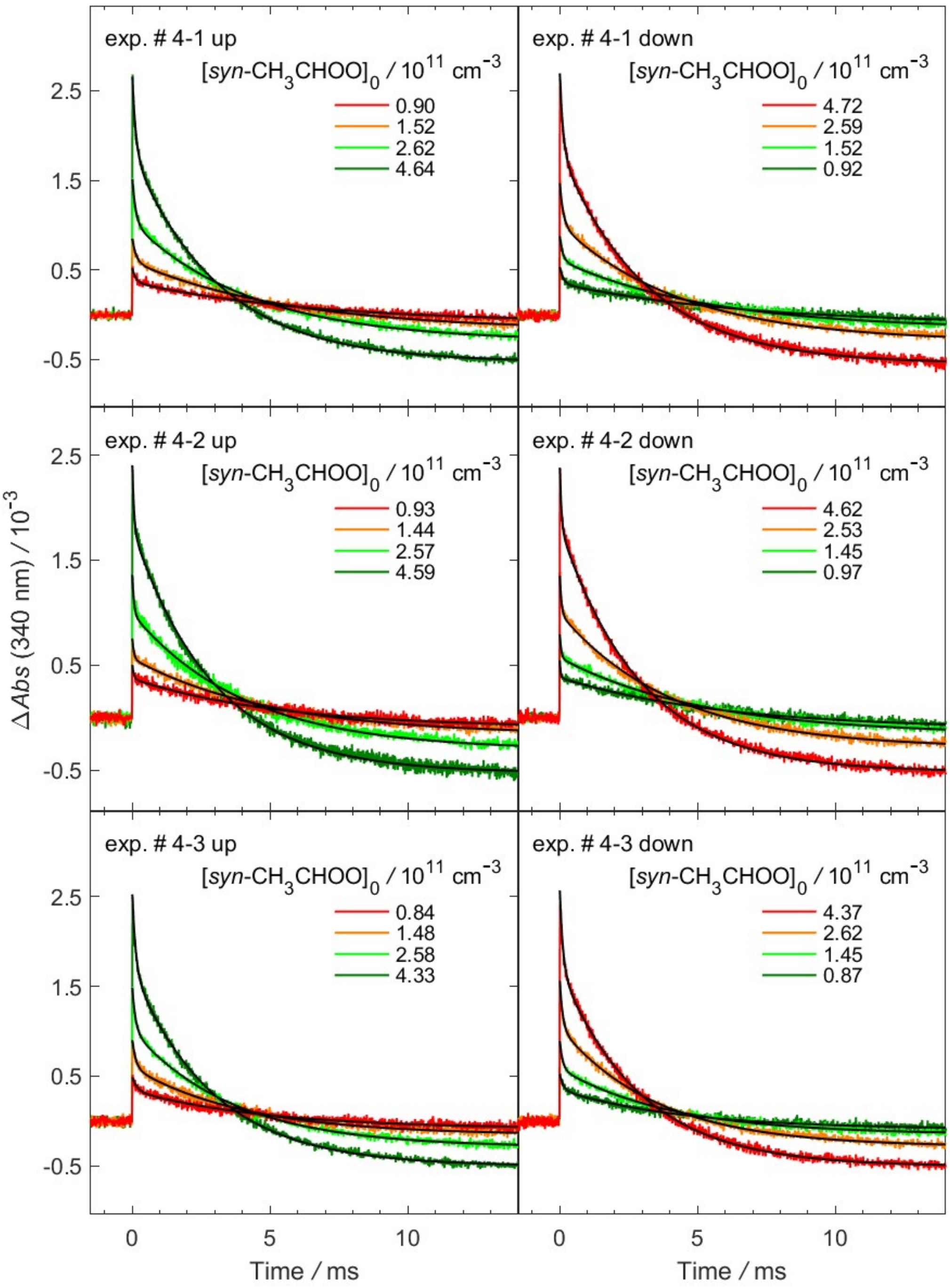

Figure S17. As Figure S9, but for different experiment sets (exp. # 4-1 to 4-3).

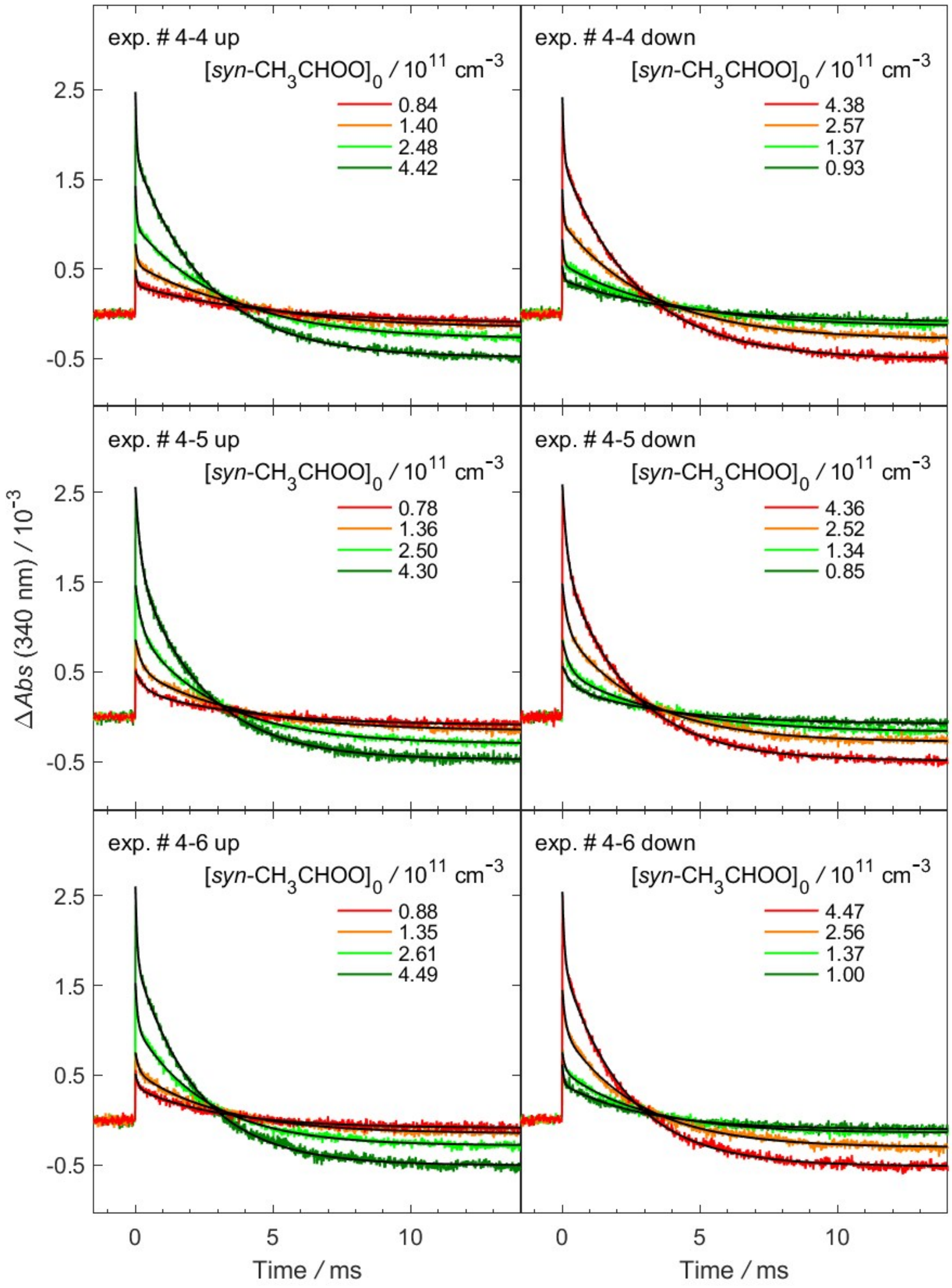

Figure S18. As Figure S9, but for different experiment sets (exp. # 4-4 to 4-6).

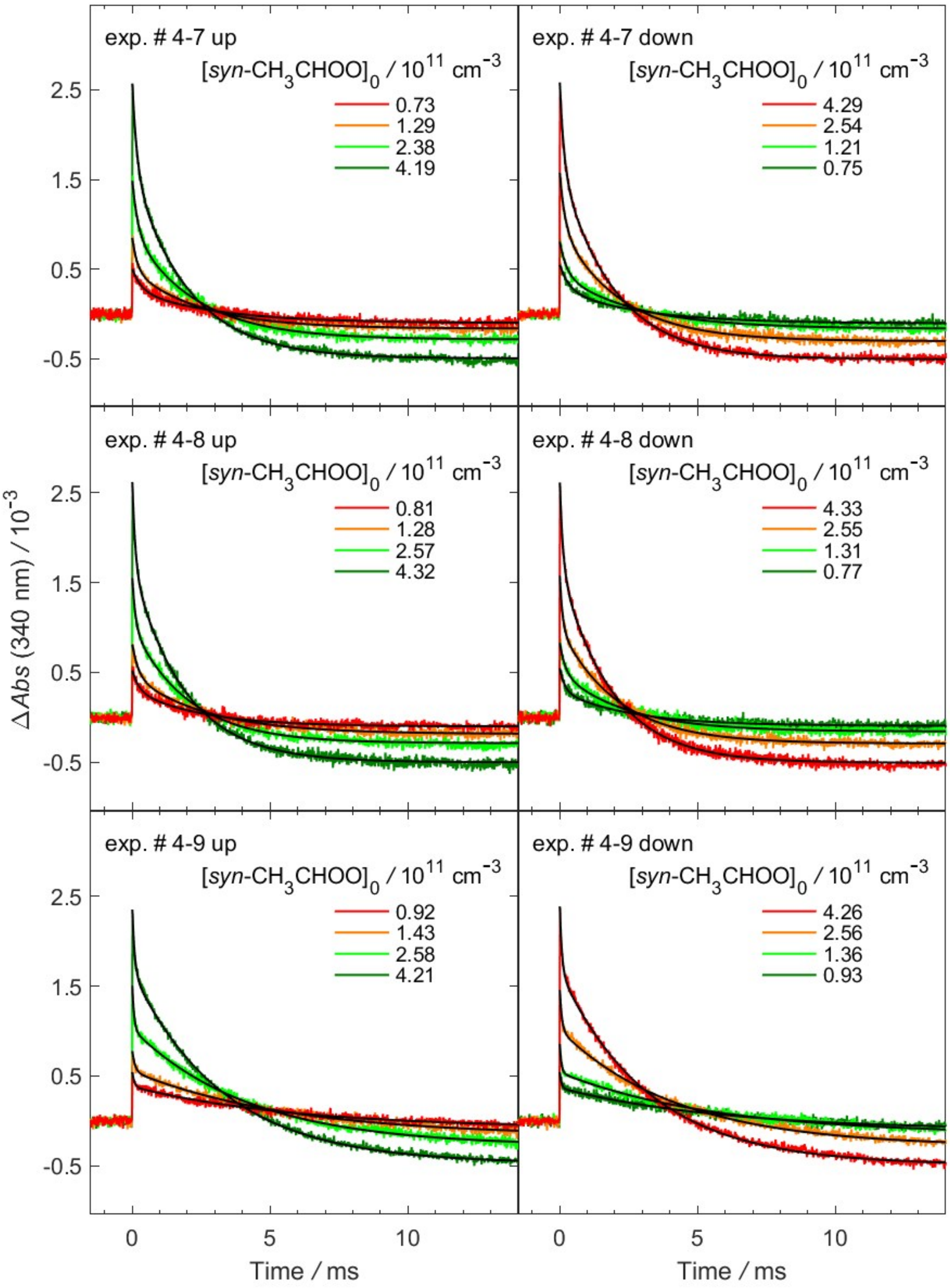

Figure S19. As Figure S9, but for different experiment sets (exp. # 4-7 to 4-9).

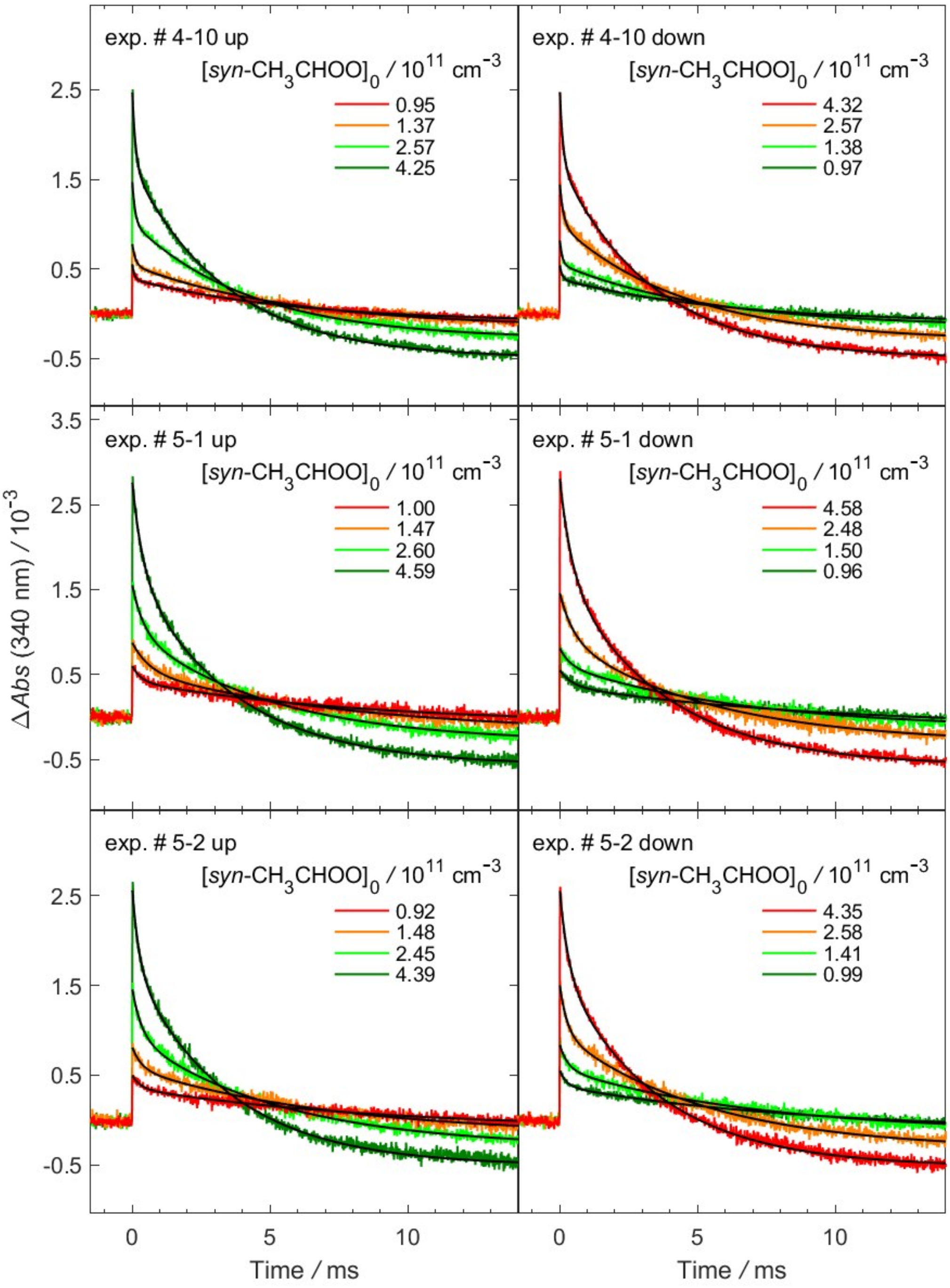

Figure S20. As Figure S9, but for different experiment sets (exp. # 4-10 to 5-2).

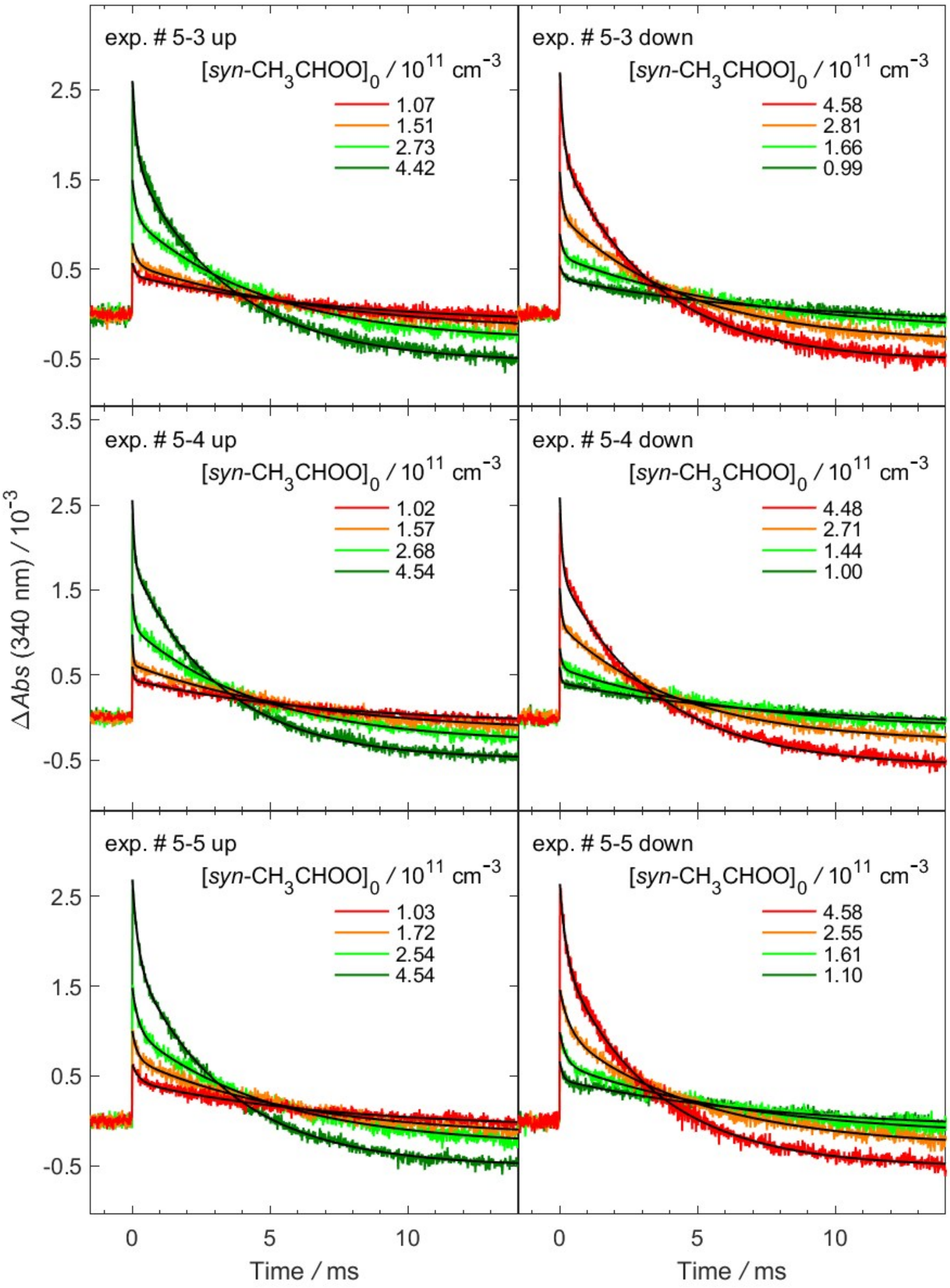

Figure S21. As Figure S9, but for different experiment sets (exp. # 5-3 to 5-5).

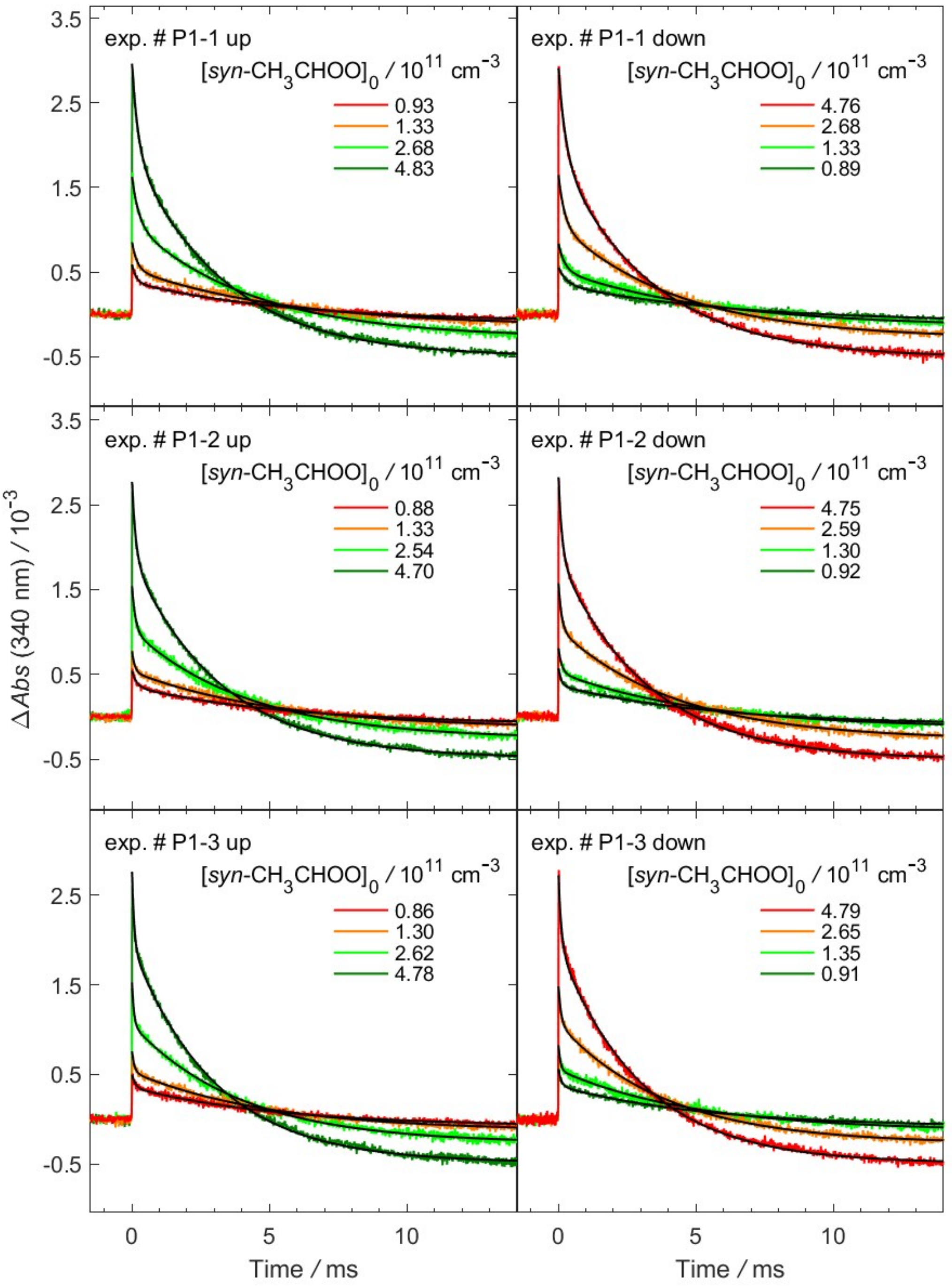

Figure S22. As Figure S9, but for different experiment sets (exp. # P1-1 to P1-3).

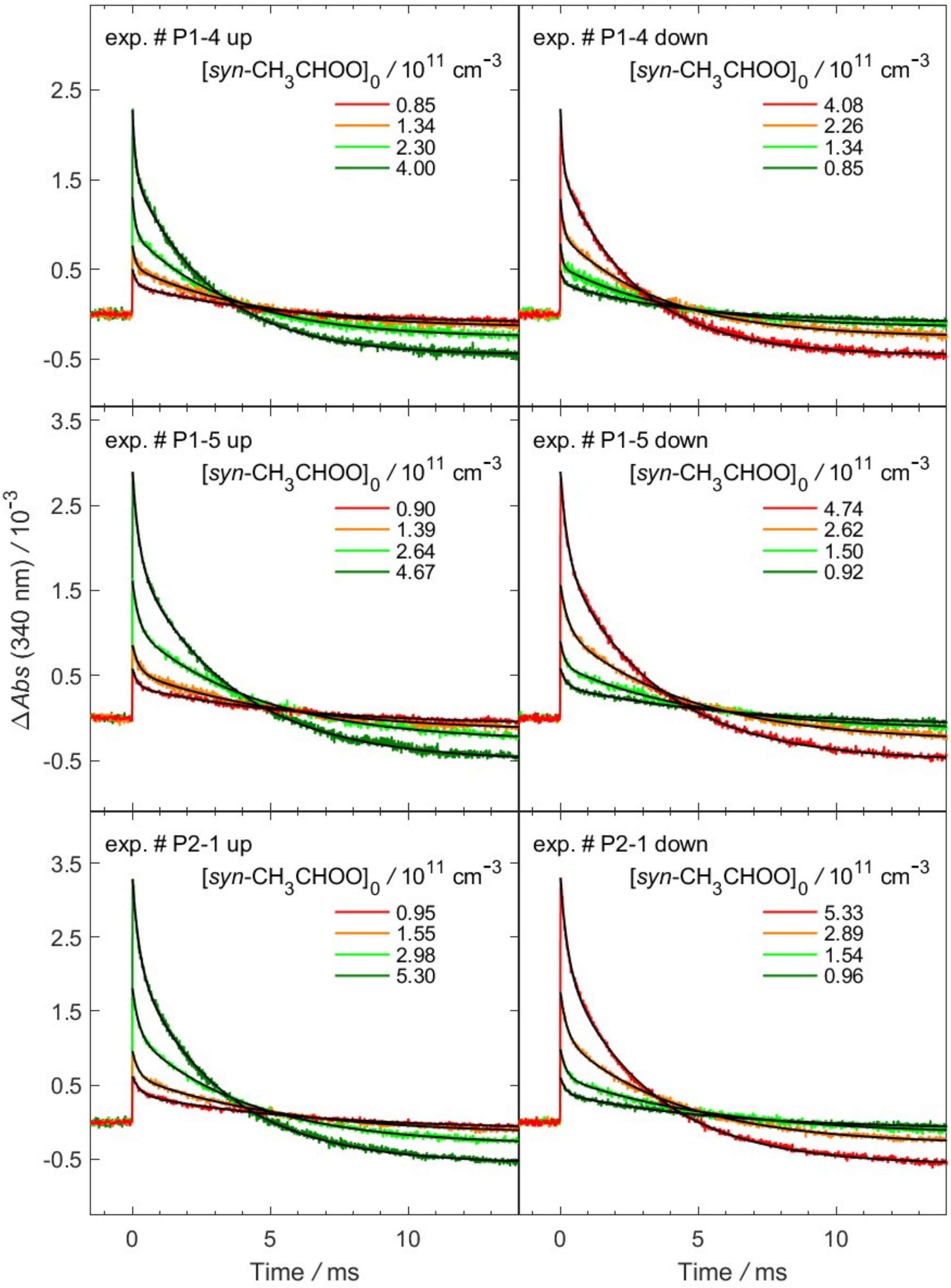

Figure S23. As Figure S9, but for different experiment sets (exp. # P1-4 to P2-1).

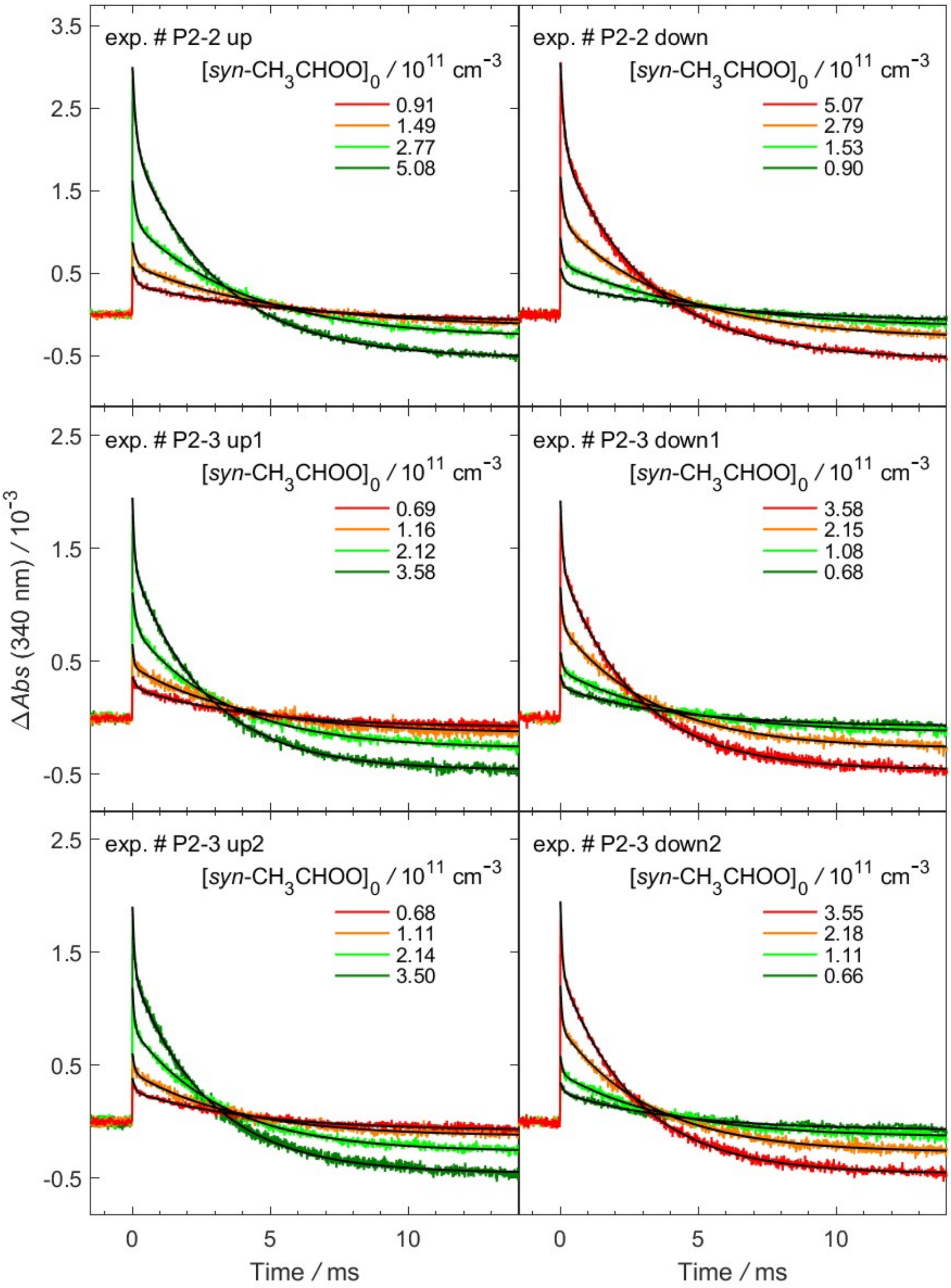

Figure S24. As Figure S9, but for different experiment sets (exp. # P2-2 to P2-3).

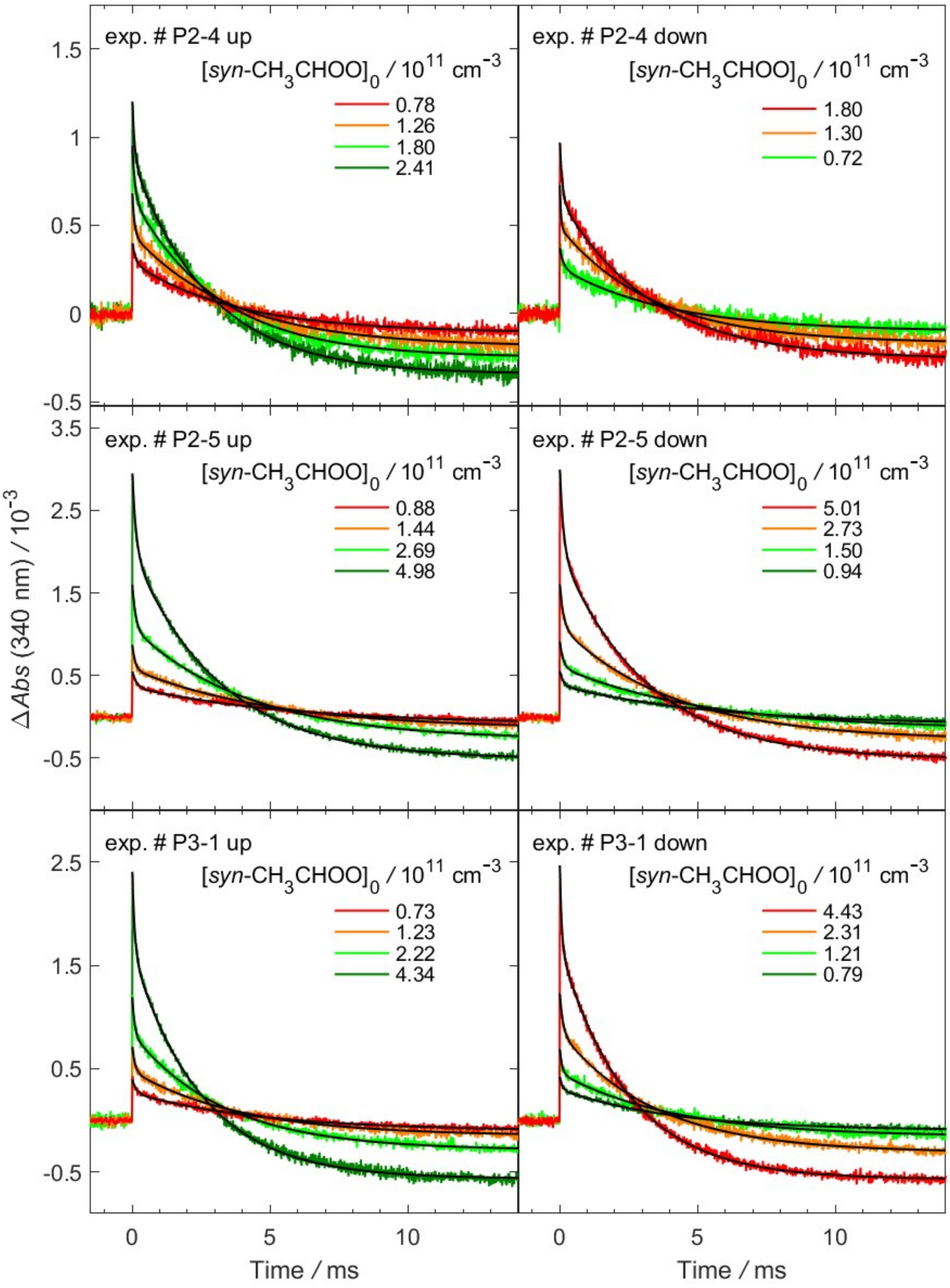

Figure S25. As Figure S9, but for different experiment sets (exp. # P2-4 to P3-1).

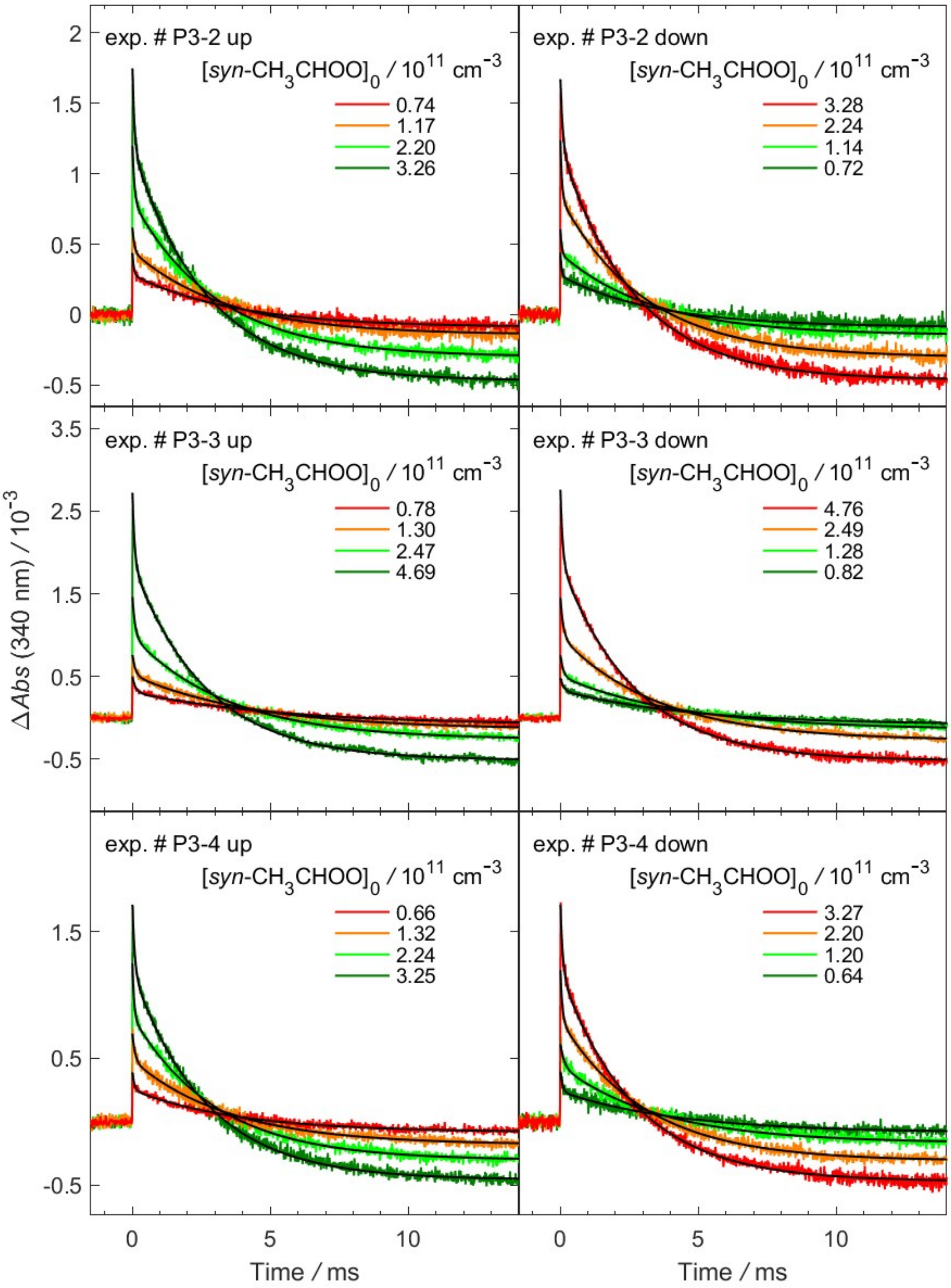

Figure S26. As Figure S9, but for different experiment sets (exp. # P3-2 to P3-4).

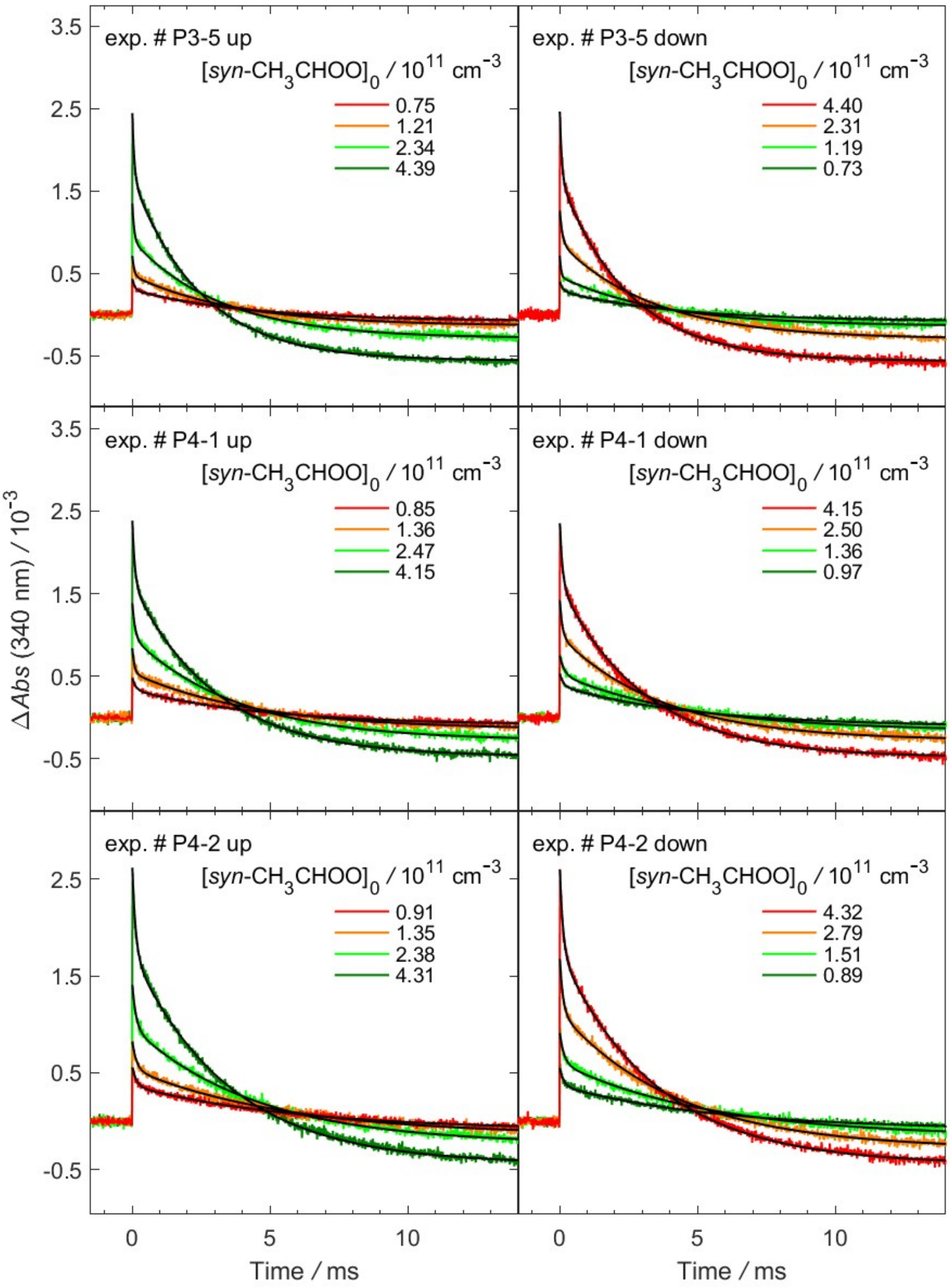

Figure S27. As Figure S9, but for different experiment sets (exp. # P3-5 to P4-2).

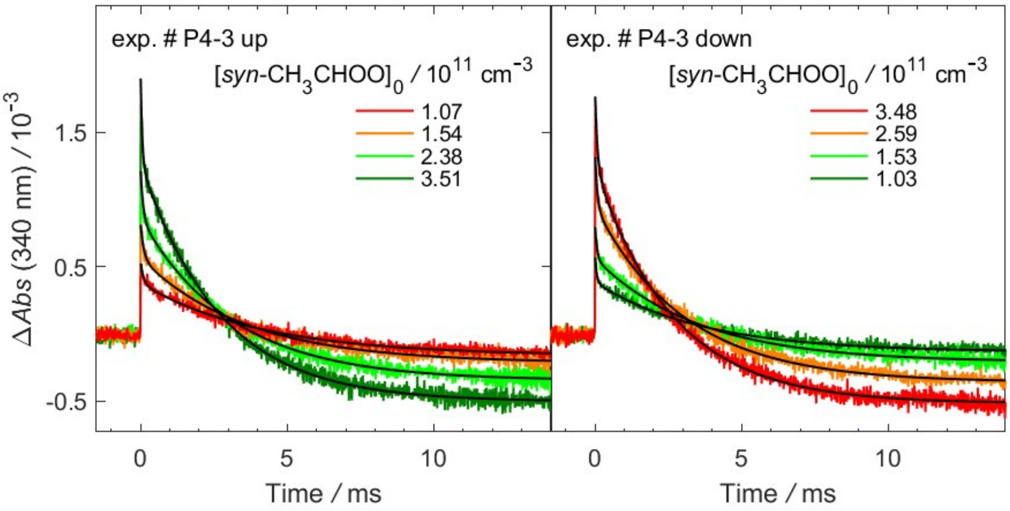

Figure S28. As Figure S9, but for different experiment sets (exp. # P4-3).

Representative time traces for the reaction of CH_3CHOO and water vapor

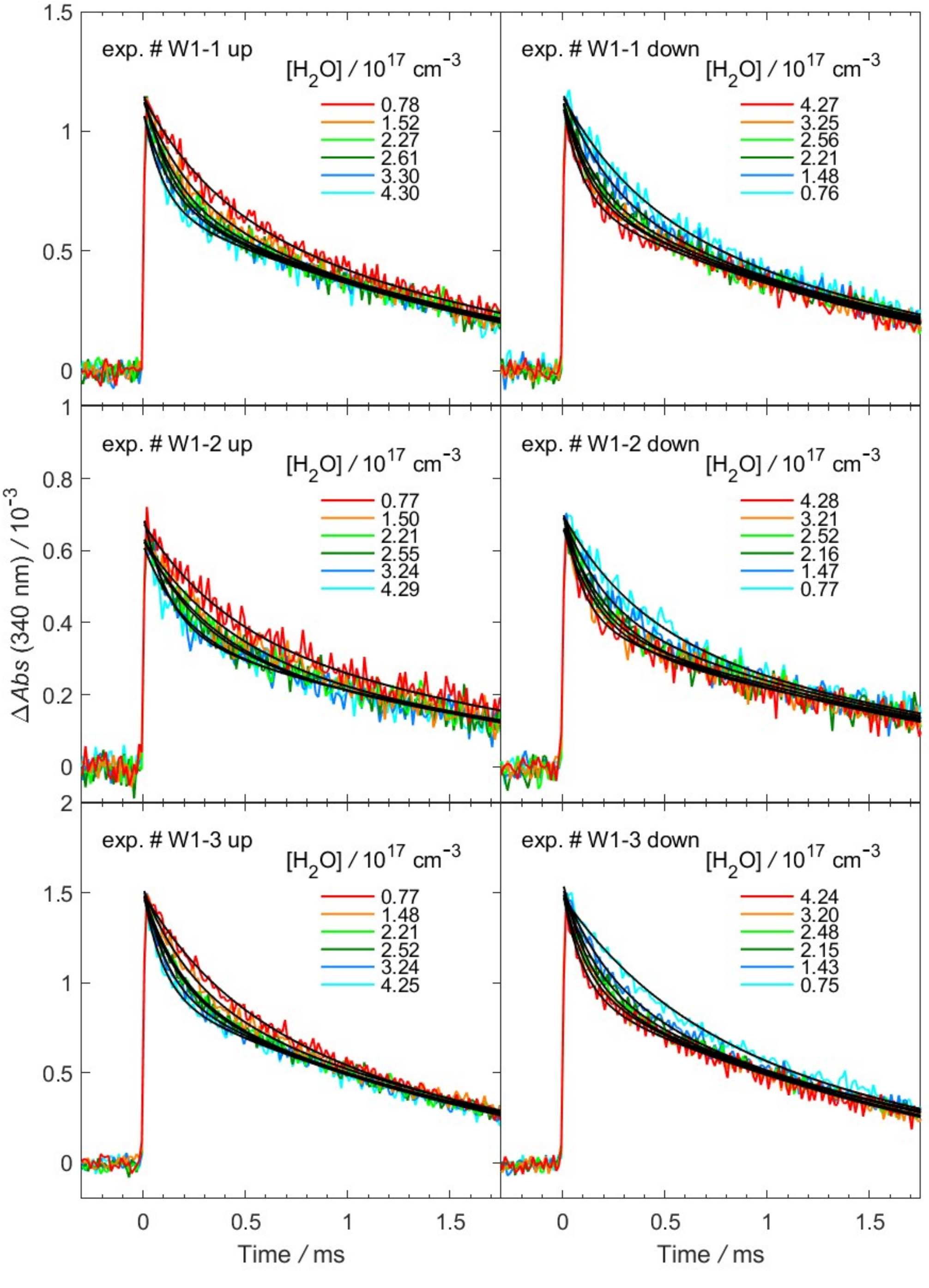

Figure S29. Representative time traces of CH_3CHOO at 340±5 nm under different [H_2O] (exp. # W1-1 to W1-3). The photolysis laser pulse sets the time zero. In each experiment, [H_2O] was scanned from the minimum to the maximum (labeled as "up") and from the maximum to the minimum (labeled as "down"). For each trace, the black line is the two-exponential fit to the signal of CH_3CHOO.

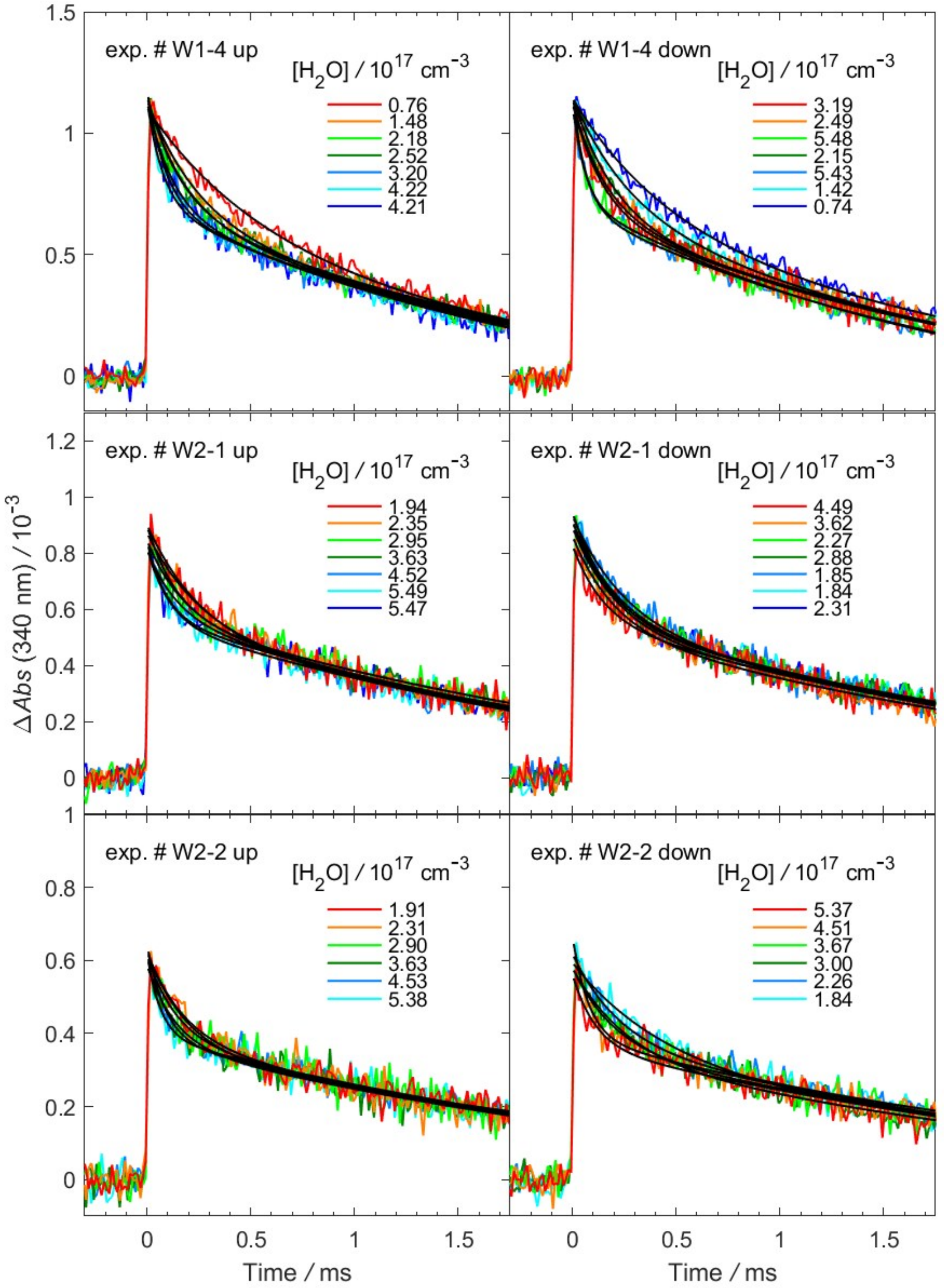

Figure S30. As Figure S29, but for different experiment sets (exp. # W1-4 to W2-2).

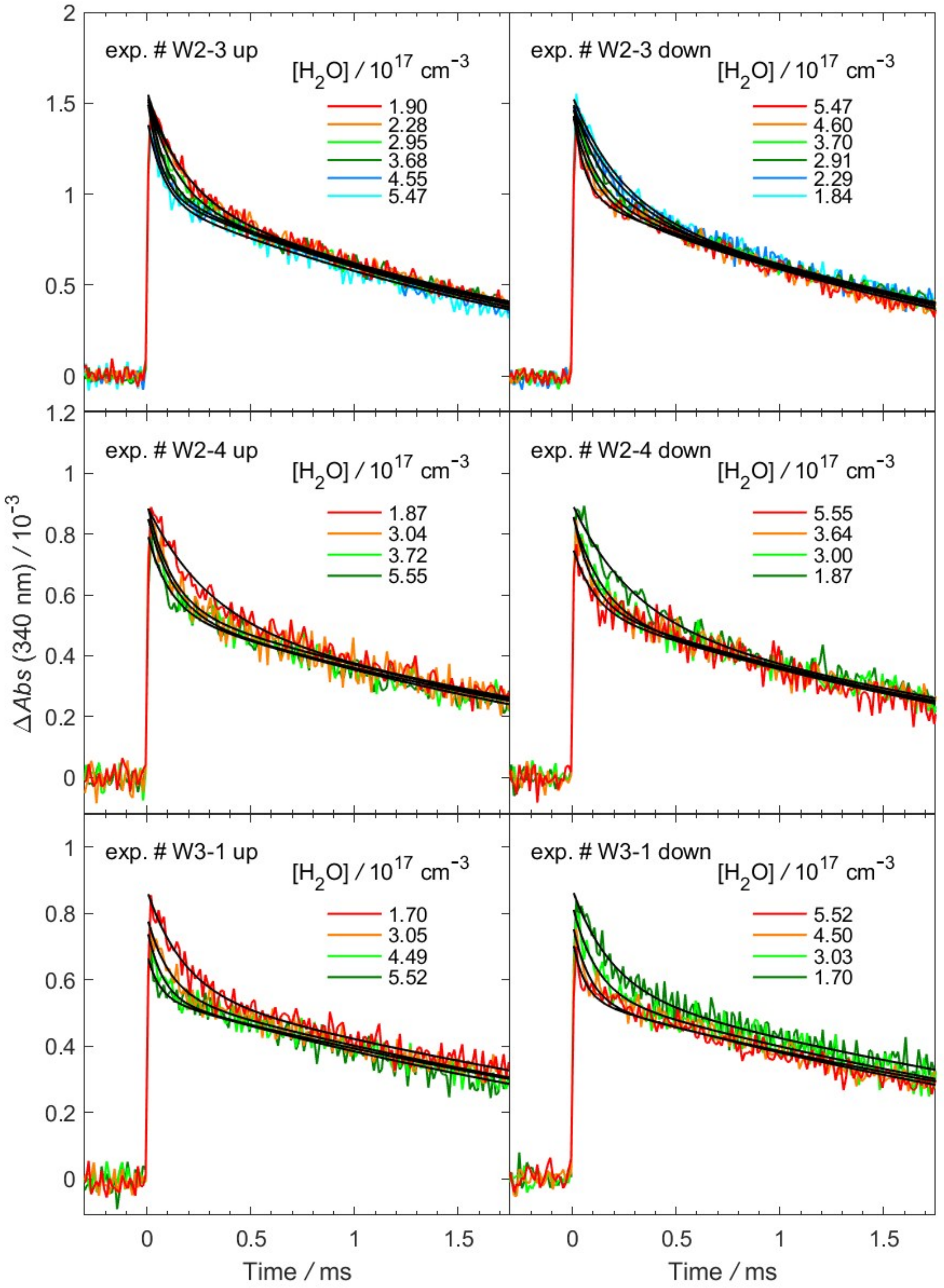

Figure S31. As Figure S29, but for different experiment sets (exp. # W2-3 to W3-1).

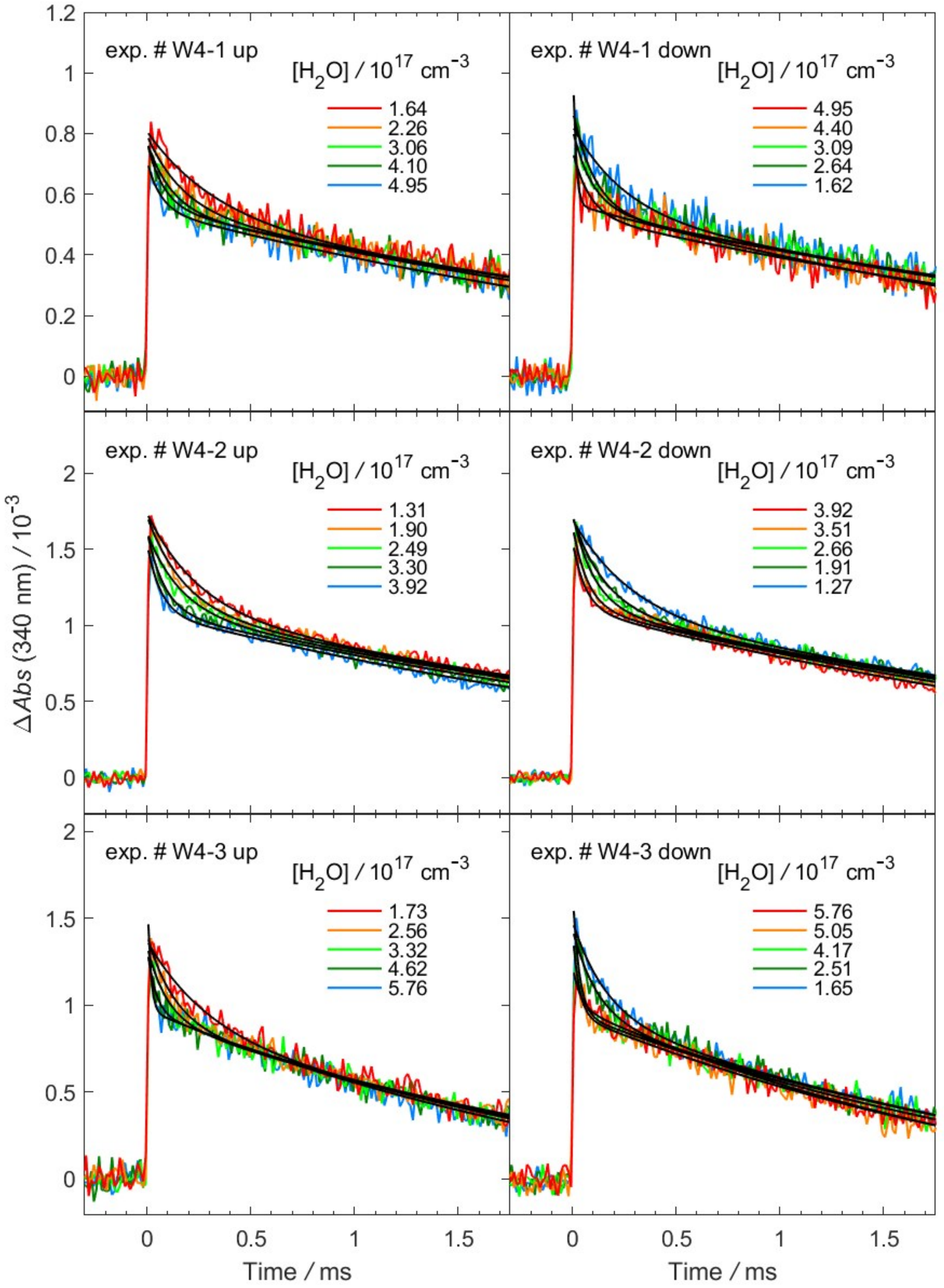

Figure S32. As Figure S29, but for different experiment sets (exp. # W4-1 to W4-3).

References

1. E. N. Fuller, P. D. Schettler and J. C. Giddings, *Ind. Eng. Chem.*, 1966, **58**, 18-27.
2. A. Blanc, *J. Phys. Theor. Appl.*, 1908, **7**, 825-839.
3. T. L. Bergman, F. P. Incropera, D. P. DeWitt and A. S. Lavine, *Fundamentals of Heat and Mass Transfer*, Wiley, 2011.
4. X. H. Zhou, Y. Q. Liu, W. R. Dong and X. M. Yang, *J. Phys. Chem. Lett.*, 2019, **10**, 4817-4821.
5. M. C. Smith, W. L. Ting, C. H. Chang, K. Takahashi, K. A. Boering and J. J. M. Lin, *J. Chem. Phys.*, 2014, **141**, 074302.
